# Antioxidant Nanozymes: From Rational Design to Biomedical Applications

**DOI:** 10.34133/research.1318

**Published:** 2026-06-30

**Authors:** Zhichao Deng, Ruofei Zhang, Yuanyuan Zhu, Chenxi Xu, Mei Yang, Lu Li, Yan Cheng, Haitao Shi, Changwei Dou, Mingzhen Zhang, Yu Xia, Kelong Fan

**Affiliations:** ^1^ Department of Gastroenterology, The Second Affiliated Hospital of Xi’an Jiaotong University, Xi’an, Shaanxi 710004, China.; ^2^ School of Basic Medical Sciences, Xi’an Jiaotong University, Xi’an, Shaanxi 710061, China.; ^3^ State Key Laboratory of Biomacromolecules, Institute of Biophysics, Chinese Academy of Sciences, Beijing 100101, China.; ^4^ Department of Thoracic Surgery, The First Affiliated Hospital of Xi’an Jiaotong University, Xi’an, Shaanxi 710061, China.; ^5^ General Surgery, Cancer Center, Department of Hepatobiliary & Pancreatic Surgery and Minimally Invasive Surgery, Zhejiang Provincial People’s Hospital, Affiliated People’s Hospital, Hangzhou Medical College, Hangzhou, Zhejiang 710053, China.; ^6^ Department of Respiratory Medicine, The First Affiliated Hospital of Xinjiang Medical University, Xinjiang,Urumqi 830054, China.

## Abstract

Antioxidant nanozymes regulate reactive oxygen species homeostasis by mimicking the core catalytic functions of natural antioxidant enzymes, including superoxide dismutase-, catalase-, and glutathione peroxidase-like activities. The clinical translation of natural antioxidant enzymes has long been hampered by inherent limitations: short in vivo half-life, susceptibility to inactivation under physiological conditions, cumbersome purification processes, high production costs, non-negligible immunogenicity, and limited targeting capacity. In contrast, antioxidant nanozymes can overcome these bottlenecks with superior structural stability, tunable catalytic activity, low preparation cost, and flexible multifunctional modification. Guided by the catalytic mechanisms of natural enzymes, researchers have established rational design strategies for antioxidant nanozymes. To date, a diverse array of antioxidant nanozymes have been developed, with promising applications in multiple biomedical fields, including inflammatory diseases, ischemia–reperfusion injury, neurodegenerative disorders, and cancer adjuvant therapy. Notably, landmark clinical progress has been achieved: The catalytic nanocrystal suspension CNM-Au8, a therapeutic candidate for amyotrophic lateral sclerosis, has advanced to phase II clinical trials. This review systematically summarizes the core catalytic mechanisms of antioxidant nanozymes, clarifies the structure–activity relationships between rational material design and catalytic performance, reviews the latest advances in their biomedical applications, and dissects the key bottlenecks restricting preclinical research and clinical translation. It aims to provide rational design principles for researchers in this field, reduce empirical trial and error in material development, and provide guidance for the further optimization and clinical translation of antioxidant nanozymes.

## Introduction

Oxidative stress, defined as the imbalance between reactive oxygen species (ROS) generation and endogenous elimination, constitutes the common pathological basis of over 200 major human diseases, including neurodegenerative disorders, cardiovascular diseases (CVDs), chronic inflammatory conditions, ischemia–reperfusion injury, and malignant tumors [1–5]. Natural antioxidant enzymes, such as superoxide dismutase (SOD) [6,7], catalase (CAT) [8], and glutathione peroxidase (GPx) [9], play pivotal roles in maintaining intracellular redox homeostasis and were once regarded as promising therapeutic candidates. Other natural redox-associated enzymes exist in biological systems, including peroxiredoxins, thioredoxin- and glutaredoxin-dependent oxidoreductases, and methionine sulfoxide reductases. However, these enzymes mainly mediate redox regulation or damage reversal and do not contribute to the primary flux of ROS elimination. Among these enzymes, SOD-based formulations have already been used clinically as adjuvant treatments for conditions including radiation-induced injury, inflammatory disorders, and ischemia–reperfusion injury. However, clinical and translational studies have revealed pronounced limitations: For example, exogenously administered SOD exhibits a circulation half-life of only several minutes in vivo, necessitating high or repeated dosing without yielding sustained therapeutic benefit [10]. Enzyme activity is also readily compromised under physiological conditions, and large-scale purification remains costly and technically demanding [10]. Moreover, the intrinsically single-function nature of native enzymes precludes synergistic multienzyme catalysis, while the lack of lesion-targeting capability and responsiveness to pathological microenvironments further results in inefficient ROS scavenging at disease sites. Collectively, these quantitative and functional constraints have substantially hindered the clinical translation and large-scale application of antioxidant enzymes [11]. Despite decades of research, no antioxidant enzyme-based therapy has been approved as a first-line clinical intervention for oxidative stress-driven diseases. Conventional small-molecule antioxidants (e.g., edaravone and vitamin C) are widely used in clinical practice, but their therapeutic efficacy in large-scale phase III trials for most chronic oxidative stress-driven diseases remains inconsistent and poorly reproducible, limited by nonspecific ROS scavenging, low catalytic activity, dose-dependent pro-oxidant risks, and insufficient lesion-targeted accumulation.

**Fig. 1.**
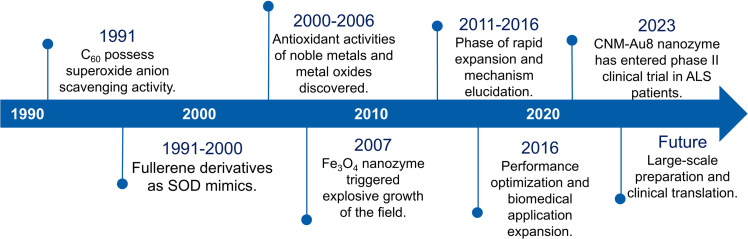
The developmental history of antioxidant nanozymes.

To address these long-standing clinical and translational limitations, researchers have spent decades exploring antioxidant nanomaterials with intrinsic enzyme-mimicking activity, culminating in the emergence of antioxidant nanozymes as a well-established new generation of artificial enzymes. The earliest evidence of this line of research dates back to 1991, when Krusic et al. [12] first discovered that C_60_ fullerenes possess superoxide anion (O_2_^•−^) scavenging activity. Following this initial discovery, a series of representative nanomaterials, including the metal oxide CeO_2_, noble metals Pt and Au, and various carbon-based systems, were successively reported to exhibit SOD- or CAT-like activities through to 2006, although these findings remained fragmented, with no systematic framework or unified terminology established for the field. A defining breakthrough arrived in 2007, when the group led by Yan et al. reported the intrinsic peroxidase (POD)-like activity of Fe_3_O_4_ magnetic nanoparticles, a finding that marked the emergence of this research field [13,14]. The period from 2011 to 2016 saw rapid expansion of the field, with over 100 antioxidant nanozymes spanning metal oxides, noble metals, carbon materials, and metal–organic frameworks (MOFs) reported, alongside systematic elucidation of the core correlations between nanostructure properties and enzyme-like catalytic activity. From 2016 to 2026, the research focus has further shifted to targeted catalytic performance optimization and biomedical application expansion, with researchers developing rational engineering strategies to enhance nanozymes’ catalytic efficiency, in vivo targeting, and biocompatibility (Fig. 1).

Benefiting from decades of iterative material design and mechanism research, antioxidant nanozymes offer multiple transformative advantages over natural antioxidant enzymes, including superior physiological stability, tunable catalytic activity, scalable low-cost production, and biocompatibility [15–19]. Moreover, material engineering enables multifunctional integration: By tailoring the composition, structure, surface chemistry, and electronic properties of nanomaterials, researchers can precisely design their catalytic behaviors and biological functions to cope with oxidative stress in complex biological contexts [20–23]. In the broad field of nanozyme research, diverse strategies have been well established to boost the intrinsic catalytic activity of nanozymes, including element doping, defect engineering, and cofactor-mediated activity modulation such as adenosine triphosphate [24,25]. These well-established approaches offer critical rational design references for the catalytic performance optimization of antioxidant nanozymes.

The advent of antioxidant nanozymes not only reflects the deep convergence of catalysis and nanotechnology but also represents a paradigm shift in biomedical engineering, moving from the concept of “substituting natural systems” to “surpassing natural systems” [26–28]. Importantly, several representative systems have already demonstrated milestone-level progress toward clinical translation: Cerium oxide nanozymes, benefiting from reversible Ce^3+^/Ce^4+^ redox cycling, exhibit sustained scavenging of O_2_^•−^ and hydrogen peroxide (H_2_O_2_) and have shown pronounced neuroprotective effects in primate and rodent models [29]. Prussian blue nanozymes, notable for their intrinsic biocompatibility and Food and Drug Administration (FDA)-recognized safety profile, have achieved efficient ROS detoxification and inflammation suppression in multiple disease models, such as myocardial ischemia–reperfusion injury, where their multienzyme-like (SOD- and CAT-like) antioxidant activities effectively alleviate oxidative stress and inflammatory damage [30,31]. More recently, single-atom nanozymes have enabled near-enzyme-level catalytic efficiency with maximized atom utilization, for example, Pt-N_4_-C single-atom nanozymes that efficiently scavenge ROS and confer robust protection against myocardial ischemia–reperfusion injury in vivo [32]. Notably, CNM-Au8, an antioxidant gold nanozyme, has advanced to phase II clinical trials for amyotrophic lateral sclerosis (ALS), representing the most advanced clinical progress of antioxidant nanozymes to date. At the nanoscale, nanozymes can mimic the active centers of natural enzymes while integrating the designability of materials science, the efficiency of catalytic chemistry, and the application-oriented perspective of biomedicine [33,34]. Consequently, their therapeutic scope now spans neuroprotection, inflammation regulation, cancer therapy, and tissue regeneration, where prolonged circulation time, multienzyme-like cascade activity, and disease-site accumulation collectively outperform conventional antioxidant enzymes in vivo [35–37]. Beyond biomedicine, antioxidant nanozymes have also shown application potential in environmental remediation and cosmetic-related fields, underscoring their interdisciplinary research value [38,39]. With the advent of rational design tools such as density functional theory (DFT) calculations and machine learning (ML), the development of antioxidant nanozymes is rapidly transitioning from empirical exploration to precision-guided construction, further accelerating their path toward clinical relevance.

However, the rapid development of this field has also brought critical unaddressed gaps that restrict further clinical translation. First, most existing studies focus on the development of single nanozyme systems with optimized in vitro activity but lack a systematic framework bridging atomic-level catalytic mechanisms, rational material design rules, and in vivo preclinical translational requirements. Second, there is still no unified international standard for the activity evaluation and biosafety assessment of antioxidant nanozymes, leading to poor comparability of research results between different laboratories. Third, the structure–activity relationships between material engineering and catalytic performance remain fragmented across different studies, and no generalizable rational design logic has been formed to guide material development, resulting in most studies still relying on empirical trial-and-error. Furthermore, systematic and guiding comprehensive reviews for this field remain lacking, with no mature full-chain roadmap from mechanism-driven design to clinical translation available for researchers.

This review aims to provide a systematic overview of the catalytic mechanisms, rational design strategies, and biomedical applications of antioxidant nanozymes. We dissect the catalytic mechanisms and rational design strategies of antioxidant nanozymes, emphasizing the correlations among electronic structure, defect engineering, surface chemistry, and enzyme-mimetic activity. We then summarize recent breakthroughs in optimizing catalytic activity, selectivity, environmental responsiveness, multifunctional integration, and cascade catalytic systems. Next, we elaborate on advances in biomedical applications, including neuroprotection, anti-inflammatory therapy, CVD management, tumor radiosensitization, tissue engineering, and disease diagnosis. Finally, we address key challenges and propose perspectives for future development. Through this comprehensive synthesis, we seek to offer researchers in nanozyme science, biomaterials engineering, and translational medicine systematic theoretical frameworks and application-focused guidance, ultimately facilitating the translation of antioxidant nanozymes from basic research to clinical practice as potent interventions for oxidative stress-related diseases.

## Catalytic Mechanisms and Characterization Techniques of Antioxidant Nanozymes

**Fig. 2.**
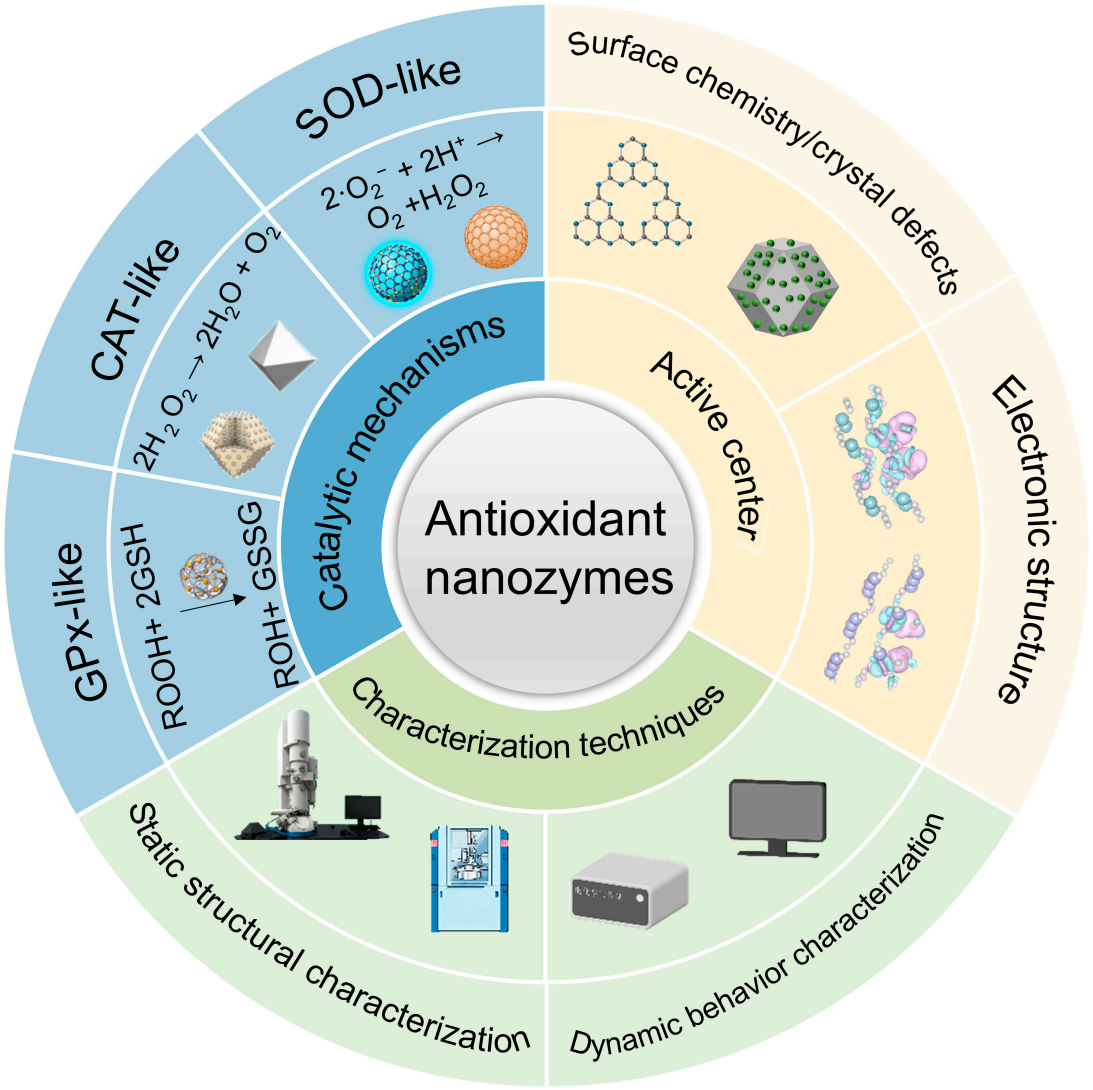
Core of antioxidant nanozymes: catalytic mechanisms and rational design.

The catalytic mechanisms and core design principles of antioxidant nanozymes form the fundamental basis for the development of high-performance biomimetic nanozymes. These mechanisms are inherently complex, involving critical processes such as electron transfer and the structural configuration of active centers [19,26]. A deep understanding and accurate simulation of these processes are essential for the rational design of antioxidant nanozymes with enhanced efficiency and stability. Accordingly, this section systematically examines the key catalytic mechanisms, strategies for constructing biomimetic active centers, advanced characterization techniques, and rational design approaches, to elucidate how these elements collectively drive progress in this rapidly evolving field (Fig. 2).

### Elucidation of catalytic mechanisms

The high catalytic efficiency of natural antioxidant enzymes relies on the precise coordination of metal cofactors while also being regulated by the conformational dynamics of amino acid residues [40–43]. These enzymes achieve efficient ROS scavenging through 2 central pathways: dismutation via sequential single-electron transfer, and multi-electron redox transfer [43,44]. Each type of enzyme-like activity follows a specific, nonrandom stepwise catalytic path driven by the reversible redox cycle of the material’s active center, and the nanozyme is not stoichiometrically consumed during ideal catalytic turnover, which is the fundamental difference between nanozymes and stoichiometric small-molecule antioxidants, and the core basis for their long-acting antioxidant effects in vivo. Inspired by these mechanisms, the design of antioxidant nanozymes has focused largely on 3 classical enzyme systems: SOD, CAT, and GPx, with the core objective of replicating the electron transfer and radical-regulation logic of natural enzymes. Such biomimicry allows the maintenance of redox homeostasis within biological systems, with the reconstruction of catalytic centers being a focal point of current research (Fig. 2).

SOD-like nanozymes catalyze the dismutation of O_2_^•−^ through sequential single-electron transfer steps, which are mainly divided into 2 material-specific catalytic mechanisms, each validated by well-established representative nanozyme systems [45–47]. The first and most widely studied mechanism relies on reversible valence-state transitions of active centers in metal-based SOD-like nanozymes, whose catalytic cycle follows 2 sequential, noninterfering half-reactions, as verified by the biomimetic dual-site framework loaded with Cu/Zn polycations (CZP@LC) developed by Lin and coworkers [48,49]. In the oxidation half-reaction, the O_2_^•−^ acts as a reducing agent, donating one electron to the high-valence Cu^2+^ active center in CZP@LC; the O_2_^•−^ is thus oxidized to molecular oxygen, while the Cu^2+^ active center is synchronously reduced to a stable low-valence Cu^+^ state. In the subsequent reduction half-reaction, another O_2_^•−^ serves as an oxidizing agent, accepting one electron from the reduced Cu^+^ active center. This step re-oxidizes the active center back to the initial Cu^2+^ state to close the valence cycle, and the reduced O_2_^•−^ combines with free protons in the physiological environment to form H_2_O_2_ [13]. The rapid and reversible Cu^2+^/Cu^+^ valence cycling in CZP@LC efficiently mediates electron transfer between O_2_^•−^ and the catalytic center, thus effectively mimicking the superoxide-scavenging activity of natural SOD. The second mechanism is electron reservoir-mediated electron transfer, which is independent of metal valence transitions and is the dominant catalytic pathway for nonmetal carbon-based SOD-like nanozymes, as typified by the activated carbon-derived carbon dots (CDs) nanozymes developed by Gao et al. [50]. For this system, surface carbonyl groups with delocalized π-electron clouds on CDs act as electron reservoirs, capturing and releasing electrons to drive the sequential single-electron transfer of superoxide dismutation. Specifically, in the oxidation step, the carbonyl electron reservoir accepts one electron from O_2_^•−^, oxidizing the O_2_^•−^ to O_2_; in the subsequent reduction step, the electron reservoir donates the captured electron to another O_2_^•−^, reducing it to form H_2_O_2_ with protons, while the carbonyl group returns to its initial state to complete the catalytic cycle. Through this alternating electron capture-release process, the CD nanozymes achieve an ultrahigh catalytic efficiency exceeding 10,000 U mg-1. The core principle for designing high-activity SOD-like nanozymes is to tune the redox potential of the active center (whether metal active sites or carbon-based functional groups) to match the redox window of the O_2_^•−^ reaction, thereby enabling both half-reactions of the dismutation process to proceed spontaneously under physiological conditions.

CAT-like nanozymes catalyze the decomposition of H_2_O_2_ into H_2_O and O_2_ via a 2-electron transfer pathway dominated by sequential proton-coupled electron transfer (PCET) steps, which are mainly implemented by redox-active metal-based nanozyme systems, with cerium- and iron-based nanomaterials as the most representative and widely studied prototypes [51–53]. The most classic CAT-like catalytic system is cerium oxide nanozymes, whose catalytic cycle strictly follows the sequential PCET process, with reversible Ce^3+^/Ce^4+^ valence cycling as the core driving force [51,52]. The entire catalytic process is completed through 3 core steps without releasing free hydroxyl radicals, consistent with the catalytic characteristics of natural CAT. First, H_2_O_2_ molecules adsorb onto the surface of cerium oxide nanozymes via hydrogen bonding or coordination with unsaturated Ce active centers; this coordination interaction polarizes the O–O bond of H_2_O_2_, and a hydroperoxo intermediate stably ligated to the Ce catalytic site is formed after a rapid proton-transfer pre-equilibrium. Second, in the rate-determining step of the entire catalytic cycle, the Ce^3+^ active center supplies 2 electrons to the bound hydroperoxo species, and the O–O bond undergoes heterolytic scission assisted by concurrent proton migration, thereby generating a high-valence Ce^4+^-oxo active intermediate and one H_2_O molecule. Third, the high-valence Ce^4+^-oxo intermediate extracts 2 electrons from a second H_2_O_2_ molecule, triggering a second round of PCET, which reduces the Ce active center back to its initial Ce^3+^ valence state, while liberating one H_2_O molecule and one O_2_ molecule to complete the closed catalytic cycle. In this system, the rapid reversible interchange between Ce^3+^ and Ce^4+^ dynamically modulates the local electron density and the abundance of oxygen vacancies at the active loci, which markedly reduces the energy barrier for H_2_O_2_ adsorption and O–O bond cleavage, thus boosting the overall catalytic turnover frequency [52]. Iron-based oxide nanozymes are another representative CAT-like system, whose catalytic mechanism follows a similar 2-electron PCET pathway with Fe^2+^/Fe^3+^ valence cycling as the core, while lattice imperfections and edge sites play a key synergistic role [53]. For iron oxide nanozymes, the edge positions with abundant lattice defects can act as electron-conduction bridges, promoting efficient charge relay from the Fe active core to the adsorbed peroxide substrate, and further optimizing the catalytic selectivity of the 2-electron transfer route. This structural feature enables iron-based CAT-mimicking nanozymes to maintain stable catalytic activity under physiological conditions, which is essential for efficient H_2_O_2_ clearance and oxidative stress alleviation in vivo. The core principle for designing high-activity CAT-like nanozymes is to optimize the valence cycling dynamics of metal active centers, regulate the concentration of oxygen vacancies and defect sites, and reduce the energy barrier of the rate-determining O–O bond heterolytic cleavage step, thereby achieving efficient and selective 2-electron catalytic decomposition of H_2_O_2_ under physiological conditions.

GPx-like nanozymes, with selenium-based nanomaterials as the most classic representative prototype, catalyze the reduction of peroxide substrates via a nucleophilic attack-initiated catalytic cycle, which relies on glutathione (GSH) as the reducing co-substrate to complete the electron transfer chain and realize catalytic turnover [54,55]. In the first phase, the reduced active center of selenium-based nanozymes (Se^2−^, reduced selenol state) acts as a nucleophile to attack the peroxide substrate, generating a 2-electron oxidized intermediate (Se^0^, oxidized selenenic acid state), while the peroxide is simultaneously reduced to the corresponding harmless alcohol or water [54]. Following this, the thiol group of an initial GSH molecule performs a nucleophilic attack on the oxidized selenium-containing intermediate, leading to a stable nanozyme–GSH adduct via a selenium–sulfur bridge and the elimination of one water molecule; this step represents the crucial linkage between peroxide reduction and GSH oxidation, and enables the subsequent regeneration of the catalytically active site [56]. The process then continues with a second GSH molecule, whose thiol group attacks the nanozyme–GSH adduct. This triggers a chalcogen–sulfur bond exchange reaction that yields oxidized glutathione (GSSG) and restores the nanozyme’s active selenol site to its original reduced Se^2−^ form, thereby closing the full catalytic cycle [56,57]. In this system, the reversible alternation between Se^2−^ and Se^0^ drives the entire electron transfer process: Electrons are harvested from the peroxide substrate by the active selenol site and subsequently handed off to GSH via the selenium–sulfur bridge [55,57]. Altogether, these mechanistic steps provide the foundation for efficient peroxide reduction and radical clearance that GPx-like nanozymes carry out in the intracellular redox environment.

In addition to the above core enzyme systems, natural organisms also have other auxiliary antioxidant pathways [58–60], which mainly participate in redox regulation and oxidative damage repair, rather than acting as the main route of ROS elimination. In contrast, the 3 enzyme systems described above (SOD, CAT, and GPx) constitute the central catalytic backbone of endogenous antioxidant defense, governing the selective and stepwise conversion of O_2_^•−^ and H_2_O_2_ into innocuous products [61]. By abstracting and replicating the electron transfer pathways, valence cycling behavior, and radical-regulation logic intrinsic to these natural antioxidant enzymes, these archetypal systems establish the theoretical foundation for the rational design of antioxidant nanozymes, thereby underpinning their development and biomedical application in nanomedicine.

### Electronic structure, defects, and surface chemistry of antioxidant nanozymes

The high catalytic performance of antioxidant nanozymes arises from the synergistic interplay of electronic structure, defect engineering, and surface/interface chemistry. These factors collectively govern the construction of enzyme-mimetic active centers and the efficiency of electron transfer [62,63]. By emulating the electron transfer pathways of natural enzymes, rational design can optimize the redox cycling kinetics of variable-valence metals, reconstruct band structures via defect states to enable multifunctional integration, and employ spatial confinement effects to precisely modulate the electron distribution at active sites. These strategies not only enable customized radical scavenging and cascade catalytic functions but also enhance catalytic stability under complex biological conditions.

The surface chemistry of antioxidant nanozymes governs their initial interactions with ROS substrates, dictating key steps of substrate recognition, adsorption, and capture that initiate the catalytic cycle. The specific types, surface density, and electronic state of the functional groups present directly shape how tightly the nanozyme binds to its target ROS, how efficiently electrons shuttle across the interface, and how readily the downstream catalytic sequence gets triggered. Moreover, these surface features are the ones steering the overall behavior of nanozymes in biological settings, influencing a wide array of critical biological attributes from biocompatibility and targeting precision to interactions with the immune system. The chemical status of these surface functional groups has a concrete and immediate impact on catalytic performance. For example, the SOD-like activity of CDs is largely attributed to their surface functionalities. Hydroxyl and carboxyl groups work to anchor O_2_^•−^ through hydrogen bonding interactions, while carbonyl groups serve as electron acceptors to facilitate the production of O_2_, effectively setting up a steady “capture-transfer” electron relay system [50]. In the case of honeysuckle-derived CDs (Hy-CDs), it has been further demonstrated that amino groups conjugated with the π-system help to stabilize radical intermediate species, which substantially cuts down the activation energy needed for the dismutation reaction to proceed [64]. Moving away from systems dominated by organic surface functional groups, we see that CeO_2_ nanozymes rely instead on metal-centered redox chemistry. Here, the core of the CAT-like activity revolves around the reversible redox cycling between Ce^3+^/Ce^4+^ oxidation states, while oxygen vacancies play a crucial part in regulating the Ce^3+^/Ce^4+^ redox equilibrium to optimize the breakdown of H_2_O_2_ [65,66]. These vacancies do not just adjust stoichiometry; they also function as electron reservoirs that speed up charge transfer between the metal centers and the substrate molecules. Collectively, these findings indicate that the electron affinity built into surface functional groups and the reversible redox switching capabilities of metal ions jointly form the foundation of biomimetic catalysis. Moreover, these characteristics represent the key regulatory parameters for exerting control over both the selectivity and the kinetics of the reaction at hand [67,68].

Defect engineering stands out as a central approach for activating otherwise inert surface sites and improving how efficiently antioxidant nanozymes shuttle electrons. At a mechanistic level, the whole idea is to introduce atomic-scale disorder that disrupts the otherwise regular, periodic electronic arrangement of the crystal lattice. Doing so brings about plenty of unsaturated coordination centers while also reshaping the material’s band profile and the kinetics of charge movement. Beyond lowering the energy barrier of the rate-limiting step, this strategy enables electronic restructuring that promotes the integration of multiple enzyme-like functions, reminiscent of the allosteric fine-tuning observed in native enzymes. Within Mn_3_O_4_/CeO*_x_* heterostructures, electron flow between CeO*_x_* clusters and Mn_3_O_4_ shifts the Ce^3+^/Ce^4+^ and Mn^3+^/Mn^2+^ balances, giving rise to paired active regions that drive a concerted SOD/CAT-type response [46]. In related work, Zhao and coworkers constructed dual-defect-rich ultrafine Pt nanowire nanozymes (Pt NM) designed to clear ROS and ease inflammatory skin disorders. In these Pt NM samples, twin-related defects impose lattice strain that moves the d-band center, which in turn adjusts both adsorption energies and the local electron density. Consequently, the active spots bind and convert assorted ROS more vigorously, enabling the material to exhibit SOD- and CAT-like activities side by side [69]. This defect-orchestrated reshaping of the electronic landscape mimics the allosteric regulation that natural enzymes rely on, giving a solid conceptual basis for tuning catalytic behavior through deliberate control over a material’s inner architecture [70].

Ultimately, the catalytic response of antioxidant nanozymes is governed by their electronic structure. The layout of the electron cloud, the exact placement of the d-band center, and the redox potential at the active site all combine to set the thermodynamic feasibility and the kinetic speed of the reaction. By carefully manipulating these electronic traits, one can directly command the catalytic output. It directly determines the electron transfer capacity between the active site and ROS substrates, the adsorption–desorption balance of reaction intermediates, and the selectivity of the catalytic pathway, which is the underlying core of rational design strategies for antioxidant nanozymes. In covalent organic framework (COF)-based artificial metalloenzymes (S-HACOF-Ru), the unique geometry of Ru–N_2_ coordination generates electron-rich regions where unpaired d-electrons facilitate the activation of oxygen intermediates by lowering the associated energy barrier [71]. In heterostructures such as Pt@CNDs, the carbonyl and hydroxyl groups of CDs chemically anchor Pt nanoparticles, establishing sp^2^-d orbital hybridization channels. This configuration allows H_2_O_2_ produced during the SOD-like reaction to decompose at Pt sites, enabling the spatially coupled decomposition of ROS intermediates [72]. This interface bonding and electron–channel co-design not only optimizes the directionality of electron flux but also enhances the selective recognition of diverse ROS by catalytic centers. This paradigm demonstrates that precise tuning of electronic distributions and site geometries at material interfaces can substantially increase enzyme-mimetic activity and durability [67].

In summary, electronic structure, defect engineering, and surface/interface chemistry represent the 3 core determinants governing the catalytic performance of antioxidant nanozymes. Electronic structure acts as the fundamental regulatory basis, which dictates reaction thermodynamics and kinetics through the d-band center position and active-site redox potential, thereby controlling electron transfer efficiency and the adsorption–desorption balance of reaction intermediates [73,74]. Defect engineering introduces atomic-scale lattice disorder to generate unsaturated coordination centers and reshape band structures, lowering rate-limiting step energy barriers and enabling multi-enzyme activity integration by mimicking the allosteric regulation of natural enzymes [75,76]. Surface/interface chemistry dominates the initial interactions with ROS, mediating substrate recognition, adsorption, and interfacial electron transfer via functional groups or reversible metal redox couples while also critically shaping the biological compatibility and stability of nanozymes in complex physiological environments [77,78].

### The critical role of characterization techniques in mechanistic elucidation

A deep understanding of the catalytic mechanisms of antioxidant nanozymes relies on multidimensional and high-precision characterization techniques. Morphological and structural analyses form the foundation. Scanning electron microscopy (SEM) and transmission electron microscopy (TEM) provide information on particle size, morphology, and dispersion, all of which directly influence the specific surface area and the efficiency of contact with radical substrates [79,80]. Dynamic light scattering (DLS) is employed to measure the hydrodynamic size of nanozymes in solution, while zeta potential analysis reveals their surface charge. The hydrodynamic size determines diffusion behavior and cellular uptake efficiency, whereas surface charge governs electrostatic interactions with charged radicals or biomolecules [81].

Understanding the chemical composition and structural identity of a material is absolutely essential to elucidate the underlying catalytic mechanisms. X-ray photoelectron spectroscopy (XPS) provides precise information on the chemical and oxidation states right at the surface, thereby identifying potential active centers, such as metal ions with specific oxidation states. It allows tracking of redox shifts and elucidation of electron-transfer pathways [82]. For the detection of surface functional groups, bond vibrations, or even hidden defects and dopants, both Fourier transform infrared (FTIR) and Raman spectroscopy are incredibly useful tools. These methods provide insights into the local chemical environment and help infer potential adsorption sites and interactions of reactive species [83]. Ultraviolet–visible (UV–Vis) spectroscopy is widely used to monitor catalytic reaction kinetics and quantify enzyme-like catalytic activity. For nanozymes built on semiconductors or noble metals, UV–Vis can go a step further by hinting at details about the band structure or plasmon resonance features, effectively linking optical features, such as plasmonic absorption, to catalytic redox behavior [84]. Nuclear magnetic resonance (NMR), particularly ^1^H NMR, provides insight into substrate changes over time and lets us spot those fleeting intermediates. This kind of information is key for piecing together a mechanistic story right down at the molecular scale [85]. Since the electrochemical fingerprint of a nanozyme directly reflects its intrinsic catalytic performance, techniques including cyclic voltammetry (CV) and chronoamperometry (CA) are widely employed for catalytic characterization. These techniques enable the precise quantification of redox potentials, determination of electron transfer kinetics, and evaluation of antioxidant performance under physiologically relevant conditions, which provide critical insights into the intrinsic driving mechanisms governing enzyme-mimetic behavior and the corresponding catalytic reaction pathways [86]. Furthermore, in situ characterization techniques are indispensable for real-time monitoring of dynamic structural and chemical evolution during the catalytic process, with in situ XPS, in situ Raman spectroscopy, and in situ electron microscopy serving as core and widely applied tools in this field [31,87]. They enable real-time observation of the surface structure, chemical shifts, and intermediate species under genuine working conditions, allowing direct observation of active-site evolution and reaction intermediates under working conditions. Looking at things from this temporal angle helps us grasp not just the “what” but also the “how” and “when” of nanozyme regulation.

Beyond the physicochemical and dynamic characterization of nanozymes themselves, the quantitative evaluation of ROS-scavenging ability and substrate specificity via targeted functional characterization techniques is a core pillar for mechanistic elucidation. As systematically summarized in the latest review, the key methods for assessing the ROS-scavenging performance of antioxidant nanozymes include electron spin resonance (ESR/EPR) spectroscopy, recognized as the gold standard for free radical detection, which achieves direct qualitative and quantitative analysis of O_2_^•−^, hydroxyl radicals, singlet oxygen, and other ROS through specific spin trapping technology; fluorescence- and chemiluminescence-based probes, which realize dynamic visualization and in situ monitoring of ROS clearance efficiency at the cellular and in vivo levels; and UV–Vis spectrophotometry-based quantitative systems, including enzyme-like kinetic assays for SOD, CAT, and GPx activities, as well as general free radical scavenging evaluation represented by 1,1-diphenyl-2-picryl-hydrazyl (DPPH) and 2,2′-azino-bis (3-ethylbenzothiazoline-6-sulfonic acid) (ABTS) assays. These functional characterization methods provide direct experimental evidence for the ROS-scavenging activity of nanozymes, clarifying their catalytic substrate selectivity and dissecting the intrinsic structure–activity relationship and catalytic mechanisms [88,89].

In summary, these characterization techniques—spanning morphology, structure, composition, chemical state, electronic properties, reaction kinetics, and noninvasive in vivo biological performance evaluation—provide a systematic toolbox for dissecting the structure–activity relationships of antioxidant nanozymes. They not only uncover the molecular mechanisms underlying efficient ROS scavenging but also lay the experimental and theoretical foundation for the rational design of materials with precisely tuned enzyme-like activity and catalytic selectivity, thereby deepening our understanding of the biomimetic relationship between artificial nanozymes and natural enzymes.

## Strategies for Rational Design

The rational design of antioxidant nanozymes is guided by principles from advanced materials science and biomimetic catalysis. These strategies include the careful selection of material types, precise control of structural and compositional features, biomimetic designs inspired by natural antioxidant enzymes, guidance from theoretical calculations, and, increasingly, data-driven approaches using ML. The overall aim is to replicate essential enzymatic functions while predicting and optimizing catalytic performance, enabling the creation of artificial antioxidant nanozymes with high efficiency, robust stability, and tunable functionalities.

### Rational design of antioxidant nanozymes from a materials perspective

The rational design of antioxidant nanozymes hinges critically on material choice, as a material’s intrinsic physicochemical traits directly govern catalytic activity, substrate selectivity, and biocompatibility. Viewed through a materials science lens, antioxidant nanozymes fall into 4 major categories: carbon-based materials, noble metal-based materials, metal oxide-based materials, and crystalline porous framework materials such as MOFs and COFs (Table 1). Carbon-based materials, spanning graphene and its derivatives, carbon nanotubes, and CDs, feature tunable electronic structures, diverse surface chemistries, and flexible configurations; these attributes let them effectively replicate the active centers and electron transfer routes of native antioxidant enzymes [90–93]. Noble metal-based materials possess distinct redox-active metal centers with versatile oxidation states and inherent catalytic breadth, achieving potent, efficient antioxidant action via fine-tuned size and facet engineering. Metal oxide-based materials exploit reversible redox cycling among lattice metal ions, supplying structural robustness and adjustable enzyme-mimetic activity for varied antioxidant uses. Crystalline porous framework materials merge the tailorability of organic backbones, the extensive porosity of framework topologies, and the redox reactivity of metal centers, establishing a distinctive and intensively studied scaffold for antioxidant nanozyme development [72,94–100]. As a whole, these 4 systems constitute the most thoroughly examined foundations for building high-efficacy antioxidant nanozymes. Thoughtful selection and structural refinement within these classes pave the way for boosting nanozyme potency, durability, and clinical impact in inflammation, ischemia–reperfusion injury, and other ROS-driven conditions.

**Table 1.** Summary of core material systems for antioxidant nanozymes

Material category	Typical representative systems	Core enzyme-mimetic activities	References
Carbon-based materials	Graphene derivatives, carbon nanotubes, CDs, heteroatom-doped carbon nanostructures	SOD, CAT, GPx-like; activity modulated via surface/electronic structure engineering	[50,64,67,90]
Noble metal-based materials	Pt, Au, Ir, Pd, Ru-based nanomaterials	SOD, CAT-like cascade; activity tuned by size and crystal facet engineering	[72,95,196]
Metal oxide-based materials	CeO_2_, MnO_2_, mixed-valence metal oxides	SOD, CAT-like; ROS scavenging via reversible metal ion redox cycling	[52,101,197]
Crystalline porous framework materials	MOFs, COFs	SOD, CAT, GPx-like cascade; active-site precise regulation via framework design	[71,100,198,199]

**Fig. 3.**
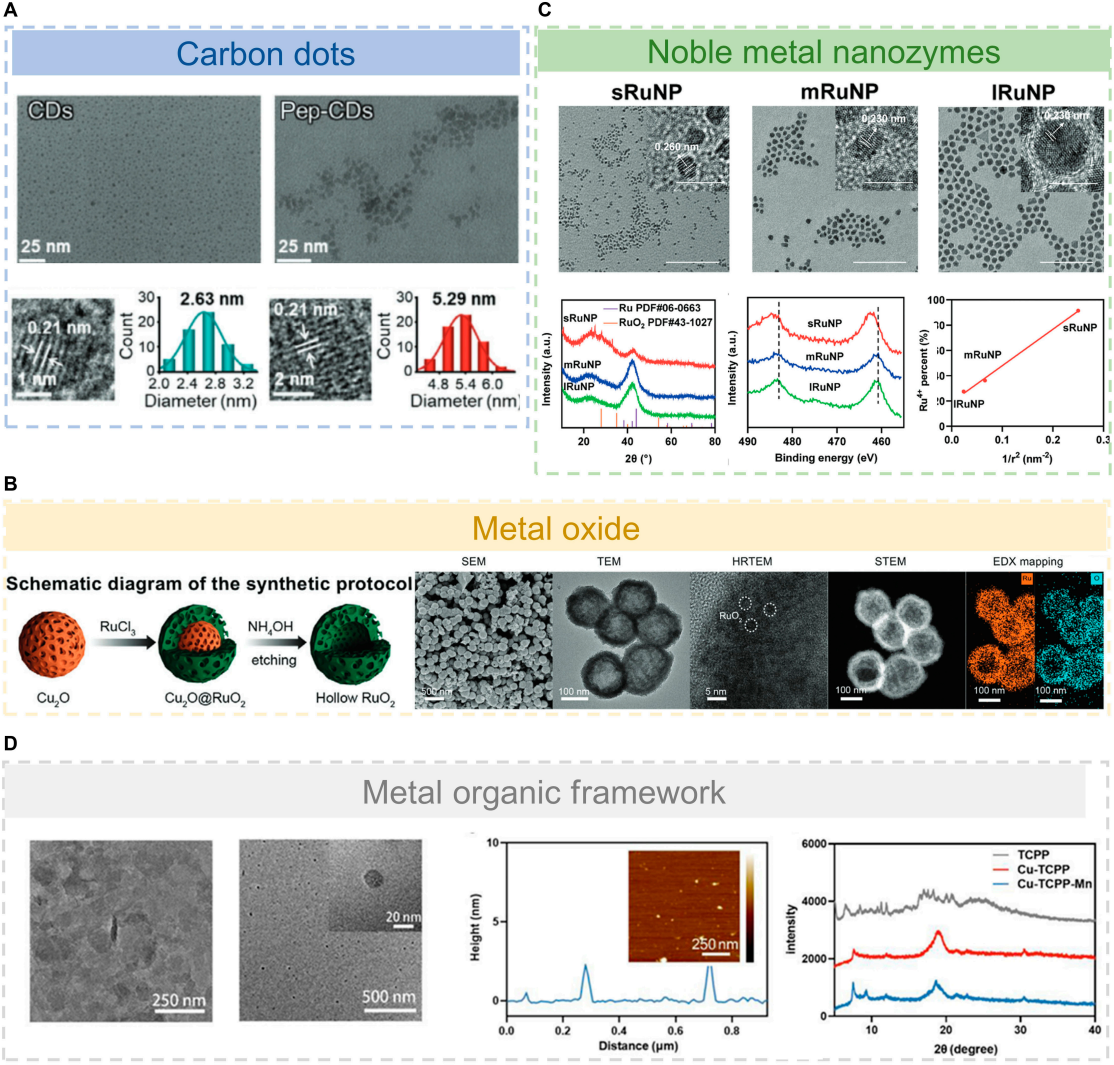
Representative materials of antioxidant nanozymes. (A) Carbon dots [90]. Copyright 2024, Wiley-VCH. (B) Metal oxide [98]. Copyright 2025, Wiley-VCH. (C) Noble metal nanozymes [95]. Copyright 2022, Wiley-VCH. (D) Metal–organic frameworks [100]. Copyright 2023, Xiang et al.

Carbon-based materials, including graphene and its derivatives, carbon nanotubes, CDs, and other heteroatom-doped carbon nanostructures, have attracted considerable attention as antioxidant nanozymes because they offer rich surface chemistry, tunable electronic structures, and good biocompatibility [50,64,67,90]. Among these, CDs are particularly attractive for several reasons: Their ultrasmall size and chemically versatile surfaces allow flexible functionalization, so you can incorporate targeting motifs and multiple enzyme-mimetic activities all in one nanoplatform. A good example is the phosphatidylserine-targeted peptide-modified CDs (pep-CDs) reported by Chen et al. (Fig. 3A). These pep-CDs keep their deep-red fluorescence and photoacoustic properties while using the CLIKKPF peptide to selectively bind phosphatidylserine on foam cell membranes. Because of this targeting ability, they accumulate more in atherosclerotic plaques, which boosts their local SOD-like antioxidant and anti-inflammatory effects [90]. It is worth noting that beyond such multifunctional integration, CDs are also compelling from a materials science angle, and their intrinsic surface structure can support remarkably high enzyme-mimetic activity. By taking advantage of the tunable surface states and well-defined carbon frameworks of CDs, researchers have made SOD-like nanozymes with specific activities above 10,000 U mg^−1^, far exceeding the 2,000 to 5,000 U mg^−1^ of natural SOD enzymes. This highlights the inherent catalytic potential of carbon-based materials. These CDs nanozymes also tend to localize preferentially in oxidation-damaged cells and mitochondria, which allows efficient scavenging of intracellular ROS and provides neuroprotection in ischemic stroke (IS) models [50]. Combined with their intrinsic stability, low toxicity, scalable synthesis, and cost-effectiveness, these merits make CDs a robust and versatile material platform for the rational design of antioxidant nanozymes.

Noble metal nanozymes, such as nanomaterials derived from platinum (Pt), gold (Au), iridium (Ir), palladium (Pd), and ruthenium (Ru), have been studied extensively as antioxidant mimics, largely because they offer intrinsically high catalytic activity, tunable redox chemistry, and pronounced size-dependent behavior. Within this family, Ru-based nanomaterials are receiving increasing attention, because of their multiple accessible oxidation states and strong ability to engage in electron transfer-mediated ROS regulation. As a case in point, Sun et al. described hollow RuO₂ nanospheres (H-RuO_2_) that possess porous shells and a high density of exposed active sites; these structural attributes markedly boosted both SOD- and CAT-like activities and, by down-regulating nuclear factor κB (NF-κB) and tumor necrosis factor-α (TNF-α) signaling pathways, helped alleviate bone loss in osteolysis models (Fig. 3B) [98]. Beyond deliberate structural engineering, particle size itself exerts a decisive influence on the antioxidant performance of noble metal nanozymes. When Ru nanoparticles (RuNPs) are scaled down to around 2 nm, the fraction of surface-oxidized Ru species rises sharply, yielding a CAT-like activity that surpasses that of native CAT as well as most previously documented Pt- and Ir-based nanozymes (Fig. 3C) [95]. This clear size-dependent trend highlights the intimate link between the surface electronic structure and catalytic output, suggesting that precise manipulation of particle dimensions and exposed crystal facets could be an effective way to fine-tune ROS-scavenging capacity.

Among metal-based antioxidant nanozymes, metal oxides stand out as a particularly substantial category, largely because the redox behavior of their metal centers directly dictates enzyme-mimicking performance. For instance, well-known examples such as CeO_2_ and MnO_2_ owe their antioxidant capabilities to the reversible redox switching of surface metal ions. In the case of CeO_2_ nanozymes, the coexistence and ready interconversion between Ce^3+^ and Ce^4+^ species provide both SOD- and CAT-like functions. Moreover, the particular crystal facets that get exposed can reshape the coordination environment around Ce sites, steering ROS activation toward distinct enzyme-mimetic routes [52]. MnO_2_-based systems work through a similar logic: They harness Mn-centered redox chemistry to imitate the natural enzyme Mn-SOD. When δ-MnO_2_ is confined within nanoarchitectures, Mn active sites become more accessible and O_2_^•−^ adsorption is facilitated, which gives a notable boost to SOD-like activity compared with free-standing MnO_2_ nanoparticles [101]. Finally, bringing together multiple redox-active elements, as seen in mixed-valence metal oxides, enables coordinated ROS regulation across different catalytic steps. This not only provides a practical way to strengthen antioxidant nanozyme activity but also widens the design window for multienzyme biomimetic systems [52,100].

Crystalline porous framework nanozymes, chiefly those built around MOFs and COFs, bring several distinct design strengths to the table for crafting antioxidant nanozymes. To begin with, the well-ordered pore architecture guarantees that metal active centers are both highly exposed and evenly dispersed throughout the structure. Beyond that, the organic backbone is tunable, which makes it possible to fine-tune the coordination microenvironment right at the active sites. Moreover, the framework’s confinement effect works to stabilize catalytic intermediates and, at the same time, lifts the efficiency of cascade-type catalytic processes. For MOF-based antioxidant nanozymes, the metal nodes function as the built-in redox-active cores that imitate natural antioxidant enzymes, while the adjustable porosity eases the transport and local enrichment of substrate molecules. Take Mn-containing MOF nanozymes as an example: They deliver impressive SOD-like performance by cycling reversibly between Mn^2+^ and Mn^3+^ oxidation states, and the surrounding framework structure markedly improves the long-term stability of these active centers under physiological conditions (Fig. 3D) [100]. Shifting focus to COF-based systems, the fully conjugated organic lattice enables fast electron movement, and the atomically dispersed sites can closely replicate the active pocket architecture seen in native antioxidant enzymes. As a result, they enable efficient cascade catalysis that mirrors SOD, CAT, and GPx activities in one material [71]. By integrating multiple redox-active components inside hybrid framework architectures, one can orchestrate cooperative ROS handling across different stages of a catalytic sequence, a strategy that not only boosts overall antioxidant nanozyme performance but also widens the possibilities for designing multi-enzyme-inspired systems.

Therefore, choosing the right material means taking a comprehensive look at catalytic pathways, biointerface behavior, structural robustness, and practical applicability. Future work ought to further investigate how surface atom arrangements, defect populations, and catalytic kinetics connect, especially the pathways of electron flow and selective radical quenching in composite material systems. Striking the right balance among catalytic output, selectivity, longevity, and biocompatibility will be essential for moving nanozymes into real clinical use. Ultimately, rational material design does more than just refine catalytic efficiency; it also enables multiple enzyme-like functions, responsiveness to changing conditions, and targeted therapeutic action, thereby providing a sturdy foundation for next-generation precision antioxidant treatments.

### Biomimetic strategies for antioxidant nanozyme design

The catalytic efficiency of natural antioxidant metalloenzymes originates from the precise organization of metal cofactors within well-defined active-site microenvironments, where coordinated metal ions participate in electron transfer and redox cycling while the surrounding ligands regulate reaction pathways and energy barriers. Rather than relying solely on the intrinsic activity of metal species, enzymatic function emerges from the synergistic interplay among metal identity, coordination geometry, spatial arrangement, and local electronic structure [102,103]. Accordingly, the biomimetic design of antioxidant nanozymes is essentially a bottom-up reverse engineering of natural enzymes at the nanoscale: By replicating the key structural elements that determine the catalytic function of natural enzymes at the atomic, molecular, and mesoscopic levels, nanozyme design breaks through the inherent limitations of natural enzymes while retaining their high catalytic efficiency and reaction specificity, which is the core logic of this design strategy. A central strategy in the rational design of antioxidant nanozymes is to engineer enzyme-like microenvironments at the atomic scale, in which metal centers are spatially isolated or paired at defined distances and stabilized by tailored coordination environments to enable efficient and selective ROS scavenging [104,105].

**Fig. 4.**
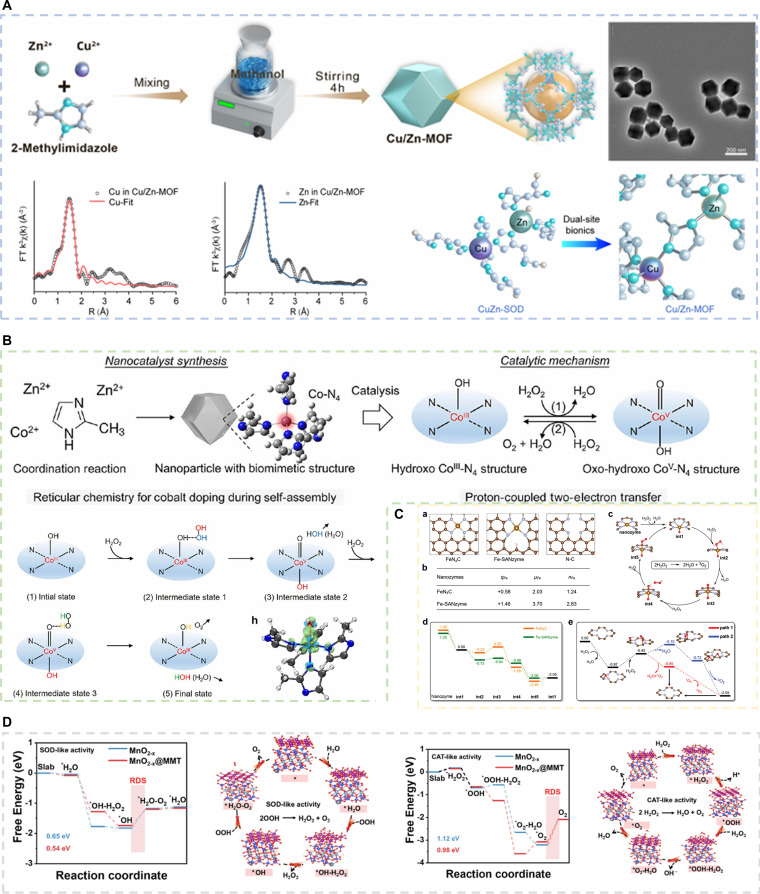
Biomimetic strategies and computational modeling. (A) Dual-site biomimetic Cu/Zn-MOF simulating the pocket microenvironment of natural enzymes [104]. Copyright 2024, ACS. (B) Co-N_4_ sites mimicking the Fe-N_4_ centers in natural CAT [105]. Copyright 2024, ACS. (C) Theoretical calculation on CAT-like activity of Fe-N_4_ sites [112]. Copyright 2022, Wiley-VCH. (D) Theoretical calculation on SOD-CAT cascade activity of Mn-based nanozymes [113]. Copyright 2025, Wiley-VCH.

Spatial arrangement biomimicry of dual active centers: precise replication of natural enzyme active pockets. The design logic of this strategy is to mimic the heteronuclear bimetallic active pocket structure of natural antioxidant enzymes, by precisely controlling the type, spacing, and coordination environment of dual metal centers, to replicate the synergistic electron transfer between bimetallic sites in natural enzymes, and realize the efficient coupling of multi-step antioxidant catalytic reactions. Accurate replication of such microenvironments has proven critical for achieving high antioxidant enzyme-mimetic activity. For instance, Huang et al. constructed a dual-site Cu/Zn-MOF that closely mimics the active pocket of natural Cu/Zn-SOD, in which Cu and Zn centers are separated by approximately 6 Å and anchored by imidazole-type nitrogen ligands analogous to histidine residues in native enzymes (Fig. 4A) [104]. This precise control over metal–metal distance and coordination geometry enables efficient SOD-like catalysis while simultaneously supporting GPx-, thioredoxin peroxidase (TPx)-, and ascorbate peroxidase (APx)-like activities, illustrating how spatially organized metal sites can facilitate multienzyme antioxidant cascades within a single framework.

Atomic coordination biomimicry of single active centers: core design for replicating the catalytic unit of natural enzymes. The design logic of this strategy is to precisely replicate the coordination environment of the metal active center in natural enzymes at the atomic level, by tuning the type of coordination atoms, coordination number, and spatial symmetry, to reproduce the electron transfer and redox cycling behavior of natural enzymes. It is the most direct and widely used biomimetic strategy for antioxidant nanozymes, and also the basis for other advanced biomimetic designs. Beyond bimetallic pairing, rational modulation of single-metal coordination environments also provides an effective route to enzyme-mimetic activity. Yang et al. exploited the structural regularity of the ZIF-8 framework to substitute Zn^2+^ with Co^2+^, generating well-defined Co-N_4_ sites that closely resemble the Fe-N_4_ motif in the heme center of natural CAT (Fig. 4B) [105]. DFT calculations revealed that this coordination environment enables proton-coupled 2-electron transfer through Co^3+^/Co^4+^ valence cycling, thereby promoting efficient H_2_O_2_ disproportionation. This example highlights how controlling coordination symmetry and electronic structure at isolated metal sites can reproduce specific catalytic functions of natural antioxidant enzymes.

Framework-confined microenvironment biomimicry: overall functional replication of natural enzyme catalytic systems. The design logic of this strategy is to go beyond replicating local active centers and simulate the overall catalytic microenvironment of natural enzymes through framework material design, including the spatial confinement effect of enzyme pockets, continuous electron transfer channels, and substrate enrichment effect, to further optimize the catalytic efficiency, reaction selectivity, and structural stability of antioxidant nanozymes. Framework design further allows microenvironment engineering beyond local coordination. By exploiting the topological tunability of COFs, Xie and coworkers precisely guided the spatial distribution of Ru centers, generating a 3-dimensional (3D) architecture with enriched electronic states near the Fermi level [71]. This tailored electronic environment lowers activation barriers for multielectron oxygen-intermediate reactions, enabling efficient cascade ROS conversion and demonstrating how extended framework geometry can modulate the electronic structure and catalytic activity of embedded metal sites. At the atomic limit, Lu et al. reported FeN_4_O_2_ single-atom catalysts that integrate key structural features of SOD, CAT, and APx within a unified coordination environment [106]. The atomic isolation of Fe centers and the precisely defined ligand field enable broad-spectrum ROS scavenging, underscoring that enzyme-like activity can be realized by encoding multiple enzymatic reaction motifs into a single, atomically isolated metal center through coordination microenvironment engineering, rather than relying on multimetal assemblies.

Collectively, these studies reveal that despite their apparent structural diversity, biomimetic antioxidant nanozymes follow common design principles centered on microenvironment engineering: precise control over metal–metal distances, coordination structures, spatial confinement, and local electronic properties. Such material-level regulation provides a unifying framework for understanding how rational nanozyme design can translate enzyme-inspired architectures into efficient and selective antioxidant catalysis.

### Guiding role of theoretical calculation

To elucidate the catalytic mechanisms behind antioxidant nanozymes, molecular simulation approaches, DFT calculations in particular, have proven to be indispensable. DFT can simulate the electronic structures, adsorption behaviors, and reaction energy profiles, thereby providing atomic-level insights into catalytic interactions [107]. Such computational work does more than just forecast activity trends; crucially, it offers a roadmap for rational structural refinement. This dual role not only speeds up the development of high-performance nanozymes but also deepens our grasp of structure–activity relationships, ultimately laying down a robust theoretical groundwork for their biomedical translation [108–110].

In studies of active sites, DFT can quantify the effects of doping or defect engineering on electronic properties. For example, Guo et al. demonstrated, through electrostatic potential and molecular orbital analyses, that pyridinic nitrogen in N-doped CDs generates electron-rich regions that enhance Fe ion chelation. Adsorption energy calculations further elucidated the mechanism for inhibiting Fenton reactions [111]. Zhang et al. showed that electron transfer from Fe to the carbon matrix in defect-engineered Fe-N_4_ sites enhances H_2_O_2_ activation, thereby improving CAT-like activity (Fig. 4C) [112]. Similarly, Liu et al. used d-band center calculations to reveal that montmorillonite supports optimize intermediate adsorption energies in Mn-based nanozymes, synergistically enhancing SOD-CAT cascade activity (Fig. 4D) [113].

With respect to catalytic pathways, DFT can identify rate-determining steps and energy barrier variations. Jiang et al. confirmed via Gibbs free energy calculations that the rate-limiting step for Co(OH)_2_ nanosheet-catalyzed H_2_O_2_ decomposition is the Co^2+^→Co^3+^ transition, with the dinuclear active centers enabling efficient dismutation through inner-sphere electron transfer [109]. In Fe–Mn dual-atom nanozymes, DFT revealed the cooperative mechanism underlying SOD/CAT and clarified the cascade reaction pathway O_2_^•−^→H_2_O_2_→O_2_ [113]. Yu et al. explained the electronic basis for the high antioxidant activity and low pro-oxidant behavior of Cu-MOFs by comparing the d-band center positions of copper-based materials [79]. For biologically derived nanozymes, Ma et al. combined XAFS and DFT to show that phosphate doping in ferritin alters Fe-core coordination to form Fe–P bonds, which enhances SOD-like activity and provides a molecular template for biomimetic design [26]. In dynamic response systems, Zhao et al. employed DFT calculations and electromagnetic field simulations to clarify the interfacial charge redistribution and plasmon-induced hot electron transfer from Co_7_Fe_3_ to ZnO under near-infrared (NIR) irradiation, which selectively activates POD-like ROS generation or SOD/CAT-like ROS-scavenging pathways, enabling intelligent ROS switching [108]. These DFT studies go beyond simply clarifying how electronic structure, coordination environment, and adsorption energetics steer the catalytic behavior of specific nanozyme system. They also supply a foundation for deriving more widely applicable design rules. In one systematic computational investigation, a detailed thermodynamic and kinetic look at O_2_^•−^ dismutation on nanomaterial surfaces yielded 2 quantitative principles that govern SOD-like activity [114]. The first, termed the energy-level principle, emphasizes that intermediate frontier molecular orbitals play a decisive part in mediating electron transfer and thereby set whether the reaction follows a highest occupied molecular orbital (HOMO) or lowest unoccupied molecular orbital (LUMO) mediated pathway. The second, the adsorption energy principle, defines specific energy windows that tip the balance in favor of selective O_2_^•−^ dismutation over competing side reactions. Follow-up experimental work on MOF systems has validated the predictive strength of these ideas, confirming that they offer real utility for computational screening and for the rational design of nanozymes that possess intrinsic SOD-mimetic activity [114]. Taken as a whole, this work constructs a unified framework that links a material’s electronic properties directly to its antioxidant function while pulling together the mechanistic threads laid out in earlier DFT studies.

On a broader level, DFT and molecular simulations now do much more than unpack the nuances of electronic structure regulation, reaction energy barriers, and interfacial dynamics. They are fundamentally moving antioxidant nanozyme design away from empirical trial and error and toward a more theory-driven framework. Such computational approaches can direct how atomic arrangements are set, how coordination environments are tailored, and how defect engineering is applied, with the goal of optimizing both the electronic structure and the spatial configuration of catalytic centers for efficient, multi-enzyme ROS scavenging. With ongoing improvements in computational methodology and the expanding reach of high-performance computing, molecular simulations are expected to play an increasingly central role in predicting catalytic activity, crafting bespoke microenvironments, or engineering smart nanozymes that can respond to physiological cues. These advances may facilitate their application in precision medicine for diseases such as Parkinson’s disease (PD), inflammatory disorders, and wound repair.

### ML-assisted design of antioxidant nanozymes

Despite substantial advances in material selection, biomimetic microenvironment engineering, and theoretical calculations, the rational design of antioxidant nanozymes remains challenged by the vast combinatorial design space and the highly nonlinear relationships between material descriptors and enzyme-mimetic performance. Parameters such as elemental composition, oxidation state, particle size, surface chemistry, defect density, coordination environment, and biological microenvironmental compatibility are often strongly coupled, making purely empirical optimization inefficient. In this context, ML has emerged as a powerful complementary tool that enables data-driven exploration of structure–activity relationships beyond the limits of intuition-guided design and conventional theoretical calculations [115]. Rather than replacing mechanistic understanding, ML serves as an integrative framework that assimilates experimental data, computational descriptors, and physicochemical features to identify key determinants of antioxidant activity and guide the rational screening of nanozyme candidates with desired catalytic profiles [116].

In the early days, ML work within the nanozyme field mainly targeted classification and activity prediction across various material systems. Wei and colleagues [116] built explainable ML models using data extracted from published studies. These models were trained to predict multiple enzyme-mimetic activities, including SOD- and CAT-like functions, and achieved high predictive accuracy. Their results also revealed that transition-metal identity and periodic trends play dominant roles in shaping catalytic behavior. Moreover, sensitivity analysis offered interpretable clues about how intrinsic material features govern antioxidant activity. This demonstrated that ML can uncover hidden correlations that are difficult to extract from isolated experiments or single-parameter studies. Alternatively, Sun et al. combined ML with data mining assisted by large language models. This sped up dataset construction and improved prediction robustness, allowing them to quantitatively predict kinetic parameters like *K*_M_ and *k*_cat_ for several antioxidant nanozymes [117]. Such integration shows how ML can bridge experimental heterogeneity and may help harmonize performance evaluation by extracting comparable descriptors from heterogeneous datasets. As a result, the reliability of nanozyme design rules gets a substantial boost.

Moving past simple activity prediction, ML was particularly effective in application-oriented nanozyme discovery—especially in scenarios where you have to simultaneously optimize multiple therapeutic constraints all at once. For instance, Zhao et al. reported the ML-assisted high-throughput screening approach for treating ulcerative colitis (UC) [118]. In that study, the ML models did not rely solely on a limited number of properties; they jointly weighed antioxidant activity, acid stability, surface charge, biosafety, and how well the material interacted with the intestinal microenvironment when evaluating candidate nanozymes. By pairing feature-importance analysis with symbolic regression, the team identified SrDy_2_O_4_ as the leading candidate, an antioxidant nanozyme that not only exhibits strong ROS-scavenging ability but also restores mitochondrial function and gut microbiota balance in vivo. This case demonstrates that ML can knit together catalytic performance with specific biological demands, making it possible to uncover therapeutically viable antioxidant nanozymes that would be tough, if not impossible, to pick out using the usual trial-and-error route.

Taken together, what these investigations show is that ML serves as a formidable auxiliary tool in antioxidant nanozyme design. It excels at pulling together high-dimensional data, speeding up the material screening process, and revealing quantitative structure–activity relationships that might otherwise stay hidden. When you layer in DFT-derived descriptors and design cues inspired by natural systems, ML opens up a collaborative route toward nanozyme development that is both more predictive and more tightly aligned with real-world applications. If applied in a reasoned and careful manner, backed by solid datasets and a firm grasp of the underlying mechanisms, ML has a real shot at reducing experimental costs, boosting design throughput, and guiding the development of next-generation antioxidant nanozymes. These would be materials with not just optimized catalytic chops but also clear-cut biomedical relevance.

## Performance Advances of Antioxidant Nanozymes

### High activity and high selectivity

High catalytic activity and reaction selectivity are 2 tightly coupled but often competing requirements in antioxidant nanozyme design. High activity ensures rapid scavenging of excessive ROS, whereas high selectivity restricts reaction pathways to avoid undesired side reactions or off-target oxidative damage. From a materials design perspective, achieving both simultaneously requires precise control over the electronic structure and local coordination environment of catalytic centers, rather than simply increasing the number of active sites [101,119–123].

**Fig. 5.**
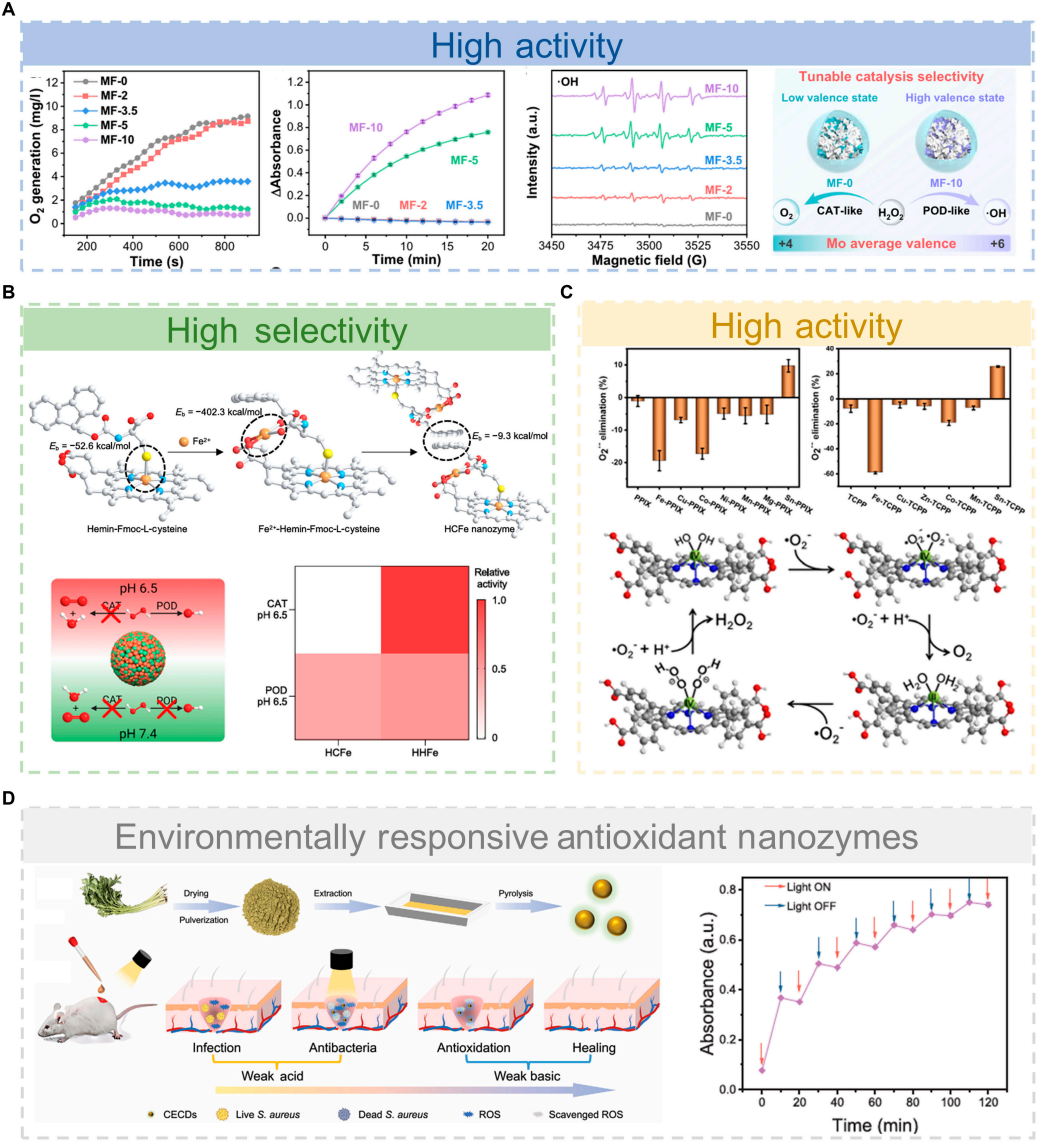
Properties of antioxidant nanozymes. (A) Antioxidant nanozymes with high catalytic activity and selectivity regulated by valence engineering [73]. Copyright 2024, Springer Nature. (B) Coordination chemistry of HCFe nanozymes enabling highly selective catalysis [124]. Copyright 2024, Springer Nature. (C) Sn-TCPP with ultrahigh SOD-like activity [121]. Copyright 2022, ACS. (D) Environmentally responsive antioxidant nanozymes [126]. Copyright 2024, Wiley-VCH.

One practical way to steer catalytic selectivity is through metal valence engineering, which directly influences electron density distribution and the resulting reaction pathways. Li et al. carried out a systematic adjustment of the average Mo oxidation state in Pluronic F127-coated MoO_3−*x*_ nanozymes by applying a mild oxidation procedure. As the Mo valence climbed from +4.64 to +5.68, CAT-like activity dropped off steadily, whereas POD-like activity became more pronounced (Fig. 5A) [73]. DFT calculations further confirmed that the valence state of Mo dictates which H_2_O_2_ activation route is favored, and consequently whether O_2_ or •OH is produced. The samples with intermediate valence numbers (MF-0 and MF-10) actually delivered the best efficiency in single-ROS reactions, which underscores the point that fine-tuning the valence is indispensable for striking a balance between high overall activity and reaction selectivity.

Moving beyond valence control, reconstructing the coordination environment at the atomic scale provides a more straightforward lever to confine the catalytic trajectory. Zhang et al. designed a heme-cysteine-Fe (HCFe) nanozyme in which the sulfur atom from cysteine binds to iron, yielding a distinctive [Fe–S] arrangement (Fig. 5B) [124]. This particular coordination pattern worked to selectively suppress CAT-like function while boosting POD-like catalysis, thereby allowing efficient generation of •OH from H_2_O_2_ under acidic pH. According to DFT analysis, the Fe–S coordination originating from cysteine feeds electrons into the Fe center, pushing the HOMO energy level outside the redox window required for H_2_O_2_ disproportionation. As a result, the second electron transfer step that belongs to the CAT pathway gets blocked. Moreover, the moderate adsorption free energy of •OH at the Fe–N_4_S site helps both with H_2_O_2_ activation and with turning over hydroxyl radicals, creating a kinetic bias that steers the process toward POD-like behavior. This case makes it clear that local coordination chemistry can essentially override the native activity of the metal, offering a robust handle for attaining highly selective catalysis [124].

In addition to tuning the same metal center, introducing alternative metal ions with distinct redox characteristics represents another effective route to enhance both activity and selectivity. Li et al. compared the SOD-like activities of metalloporphyrins and found that tin porphyrin (Sn-TCPP) exhibited markedly higher activity than conventional Mn-based analogs (Fig. 5C) [121]. When incorporated into Sn-PCN222 MOF nanozymes, the material maintained high catalytic efficiency alongside thermal and acid stability. Mechanistic studies attributed this performance to the Sn^3+^/Sn^2+^ redox cycle and the formation of J-aggregates in acidic environments, which is associated with enhanced SOD-like activity. These results indicate that subtle differences in metal electronic configuration and aggregation state can fundamentally reshape catalytic outcomes [121].

Taken together, these findings indicate that high activity and high selectivity in antioxidant nanozymes are not 2 separate, mutually exclusive traits. Instead, they can be pursued and achieved simultaneously through the deliberate manipulation of metal valence states, the engineering of coordination environments, and the controlled utilization of intrinsic metal redox chemistry. Design approaches that operate at this materials level offer a tangible, workable foundation for building nanozymes whose catalytic behavior is both predictable and tunable. Such control is critical if these nanomaterials are to function reliably in the messy, multifaceted context of biological systems.

### Environmentally responsive antioxidant nanozymes

In the setting of complex pathological microenvironments, oxidative stress is highly dynamic rather than static—it shifts continuously over the course of disease, with variables like ROS levels, pH, enzyme activities, and the local redox milieu all in constant flux. Consequently, antioxidant nanozymes that work in a constitutive, always-on manner tend to run into a timing mismatch: Their activity windows rarely align perfectly with the evolving pathological state. As a result, this may lead to suboptimal therapeutic effect or, in more severe case, unintended meddling with physiological redox signaling. From the perspective of rational design, the whole point of environmentally responsive antioxidant nanozymes is to tether catalytic behavior directly to the biochemical cues that arise locally, thereby allowing the enzyme-mimicking activity to be tuned dynamically, both spatially and temporally [81,125,126].

On a materials science level, this kind of responsiveness is generally accomplished not by installing wholly new catalytic centers but rather by using external or local stimuli to tweak the material’s electronic structures or to modulate how easily the active sites can be accessed. A representative case comes from work by He et al., who prepared celery-derived CDs, referred to as CECDs, that display a dual response to both pH and light (Fig. 5D) [126]. In this system, ambient pH and photoexcitation cooperate to remodel the surface electronic states and charge distribution of the CECDs, effectively toggling the redox role and availability of the very same active sites. When the environment is acidic, as in an infection setting, these CECDs exhibit light-triggered OXD-like activity, selectively producing bactericidal ROS. As the wound microenvironment gradually moves toward neutral or slightly alkaline pH values, shifts in protonation states and alterations in the available electron transfer pathways suppress further ROS generation and instead steer the same carbon-based active centers toward antioxidant reactions, facilitating ROS scavenging and tissue repair. This study makes it clear that a material’s intrinsic properties can be harnessed to reversibly switch catalytic pathways in direct response to changing environmental conditions.

The same conceptual thread runs through pH-activated nanozymes intended for inflammatory lesions, where local acidosis furnishes a reliable pathological hallmark. Jiang and coworkers reported platinum–sulfur pre-nanozymes (Pt_5.65_S) that remain catalytically silent under normal physiological conditions but undergo conversion into multienzyme-active Ptzymes once they encounter the acidic microenvironment of inflammation [127]. That conversion simultaneously activates SOD- and CAT-like cascade reactions and liberates H_2_S gas, showing that environmental responsiveness can be encoded by means of controlled structural reconfiguration rather than by permanently boosting activity. Critically, such designs restrict antioxidant function to diseased tissues, thereby keeping interference with redox homeostasis in healthy regions to a minimum.

Beyond pH responsiveness, enzymatic and physical stimuli can also be exploited to achieve localized activation. Xu et al. constructed metal-phenolic nanozyme hydrogels (TM/BHT/CuTA) that respond selectively to matrix metalloproteinases (MMPs), triggering the release of CuTA nanozymes at sites of tissue remodeling [128]. The released nanozymes then scavenge multiple ROS species while regulating macrophage polarization. In a complementary approach, Zhang et al. designed polydopamine-based nanozyme microneedles in which NIR light induces localized photothermal effects, enhancing microcirculation and activating antioxidant catalysis at inflamed skin sites [129]. In both cases, responsiveness is achieved by coupling catalytic activation to disease-associated biochemical or physical signals.

Taken together, these examples reveal a shared design principle for environmentally responsive antioxidant nanozymes: Catalytic activity is not maximized globally but is conditionally unlocked through microenvironment-triggered modulation of active-site structure or electronic state. This adaptive strategy enables precise spatiotemporal matching between antioxidant function and pathological demand, providing a practical route to improve therapeutic specificity while preserving physiological redox balance [128,129].

### Multifunctional integration

Multifunctional integration in antioxidant nanozymes is not just about integrating multiple capabilities into one nanomaterial. Instead, from a rational design standpoint, it emphasizes the coordinated coupling of enzyme-mimetic ROS regulation with extra functions, such as imaging, targeting, and therapeutic modulation, through a unified material and structural framework. This kind of integration enables nanozymes to function as programmable platforms that synchronize catalytic activity with diagnosis and intervention in complex pathological microenvironments [130,131].

**Fig. 6.**
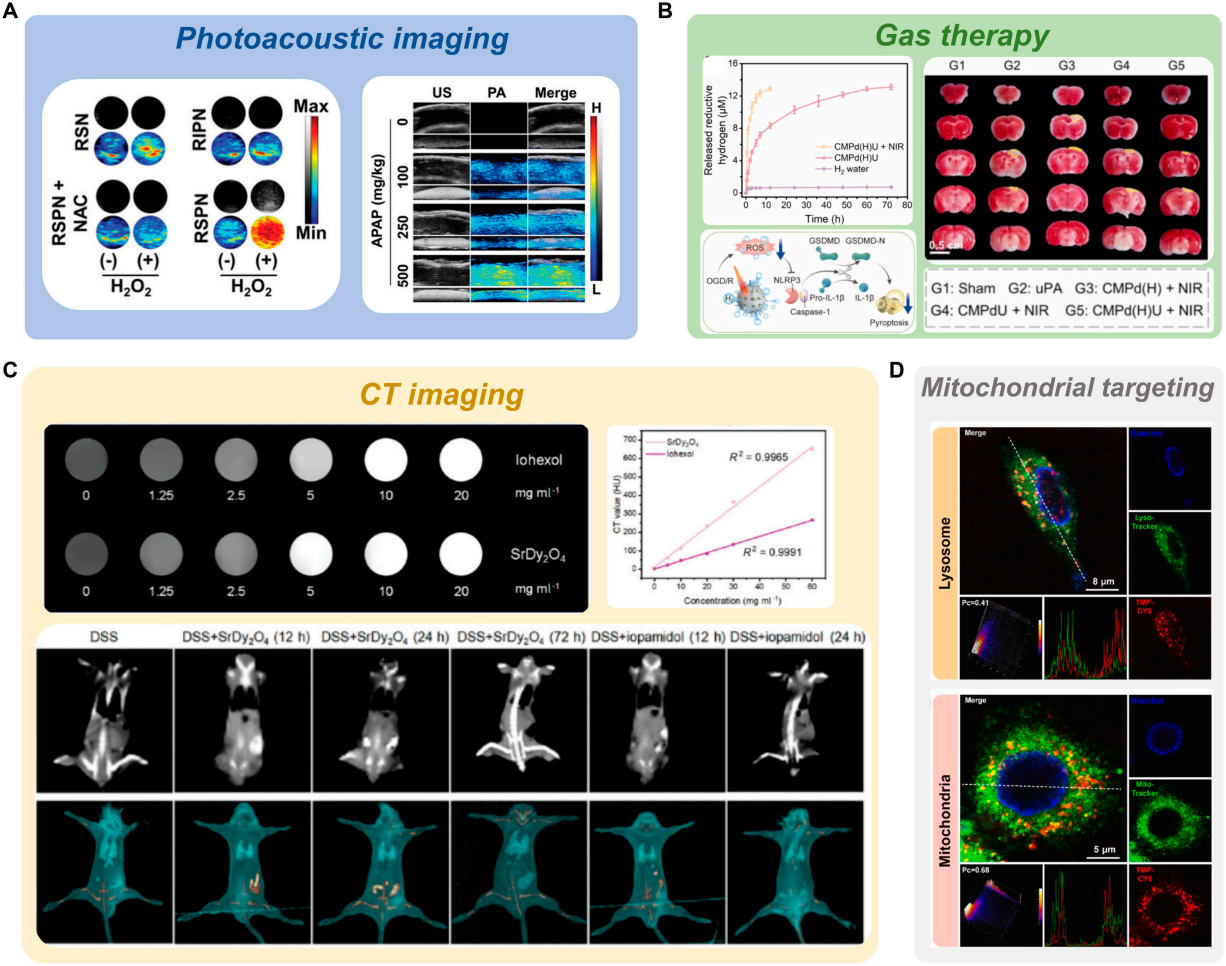
Multifunctional integration of antioxidant nanozymes. (A) ROS-responsive ceria nanozyme probe integrating photoacoustic imaging and ROS scavenging for theranostic application [132]. Copyright 2022, Wiley-VCH. (B) Multifunctional nanozyme platform with targeted drug delivery, sustained ROS scavenging, and neuroprotection for ischemic stroke therapy [133]. Copyright 2025, Elsevier. (C) Acid-stable SrDy_2_O_4_ nanozyme integrating ROS scavenging, intestinal barrier repair, gut microbiota regulation, and x-ray imaging for ulcerative colitis management [118]. Copyright 2025, Wiley-VCH. (D) Microenvironment-responsive metal-phenolic network nanozyme targeting mitochondria to integrate ROS scavenging, pyroptosis inhibition, and extracellular matrix regeneration for intervertebral disc degeneration therapy [134]. Copyright 2022, Elsevier.

A typical approach is to combine ROS scavenging with diagnostic imaging, directly linking catalytic activity to lesion visualization. Wu et al. developed a ROS-responsive ceria nanozyme-based probe that merges photoacoustic imaging with antioxidant catalysis (Fig. 6A) [132]. When exposed to high ROS levels, the ceria nanozyme both scavenges ROS and triggers gas generation, which amplifies the photoacoustic signal. In this design, imaging is not independent of catalysis; instead, it arises directly from ROS-triggered catalytic behavior. This illustrates how material responsiveness can integrate diagnosis and therapy within a single platform [132].

Beyond imaging integration, multifunctional nanozymes can be engineered to coordinate catalytic ROS scavenging with therapeutic delivery and tissue protection. Wei and colleagues [133] reported a nanozyme system for IS that integrates targeted delivery, sustained antioxidant activity, and neuroprotection (Fig. 6B). In this platform, Pd nanoparticles provide intrinsic SOD- and CAT-like activities to catalytically break down superoxide and H_2_, while lattice-stored H_2_O_2_ is gradually released to selectively quench highly reactive hydroxyl radicals during ischemia–reperfusion. The coexistence of enzyme-mimetic catalysis and molecular antioxidant release enables prolonged, broad-spectrum ROS suppression beyond the thrombolysis window. Through gas-assisted thrombolysis combined with continuous ROS scavenging, the platform addresses both vascular obstruction and oxidative injury. This example highlights how multifunctional integration can align nanozyme catalysis with disease-specific therapeutic demands, rather than just functioning as a generic antioxidant.

More advanced designs further extend multifunctionality to encompass biological regulation and real-time monitoring. Zhao et al. constructed SrDy_2_O_4_ nanozymes with acid-stable structures that simultaneously scavenge ROS, repair intestinal barrier integrity, regulate gut microbiota, and enable x-ray imaging for noninvasive monitoring in UC models (Fig. 6C) [118]. Similarly, Zhou et al. developed a microenvironment-responsive metal-phenolic network platform that targets mitochondria to scavenge ROS, thereby suppressing ROS-driven pyroptotic signaling and mitigating oxidative damage-induced extracellular matrix degradation, ultimately promoting matrix regeneration (Fig. 6D) [134]. In these systems, multiple functions are tightly linked through material composition and microenvironment responsiveness, allowing coordinated regulation of oxidative stress and downstream pathological processes.

Collectively, these studies demonstrate that effective multifunctional antioxidant nanozymes rely on the rational integration of catalytic activity with complementary biological and diagnostic functions through unified material design. Rather than increasing complexity unnecessarily, multifunctional integration enhances therapeutic precision by synchronizing ROS regulation with targeting, monitoring, and tissue modulation, thereby expanding the practical utility of antioxidant nanozymes in multifactorial disease settings [135].

### Cascade multi-enzyme systems

Cascade multienzyme antioxidant nanozymes aim to mimic the mechanisms of natural antioxidant defense system. In these systems, several enzyme-mimicking activities run one after another, and this helps to eliminate ROS in an efficient and targeted way. For example, take a SOD-CAT cascade. First, SOD-like activity converts O_2_^•−^ radicals into H_2_O_2_. Then CAT-like activity decomposes that H_2_O_2_ into H_2_O and O_2_, so harmful intermediates do not build up much. In biological systems, this high efficiency comes from substrate channeling. That happens inside multienzyme complexes that are well arranged in space, and there, reaction intermediates move quickly from one active site to the next. To put this idea into nanozyme design, we need to go beyond simply combining different enzyme-mimetic activities. We also have to control how they are placed in space and how they interact. This is key for efficient and sequential ROS conversion [72,136–138].

One key design strategy is to link cascade catalysis with a switching system that responds to the microenvironment. Then, the main enzyme function can change based on the disease stage. Liu and coworkers [139] developed sulfur quantum dot nanozymes. These can switch their catalytic activity in a dynamic way when used in infectious bone defect models. In the early infection stage, these nanozymes show SOD- and POD-like cascade activity, and that helps to produce ROS that can kill bacteria. Then, as the local environment gets less acidic while healing goes on, the catalytic behavior changes to SOD- and CAT-like activity. Therefore, it efficiently scavenge ROS and also polarize macrophages toward the M2 phenotype. This work shows that cascade nanozymes can add time-based control into multienzyme teamwork, and they do this without needing complex composite structures.

**Fig. 7.**
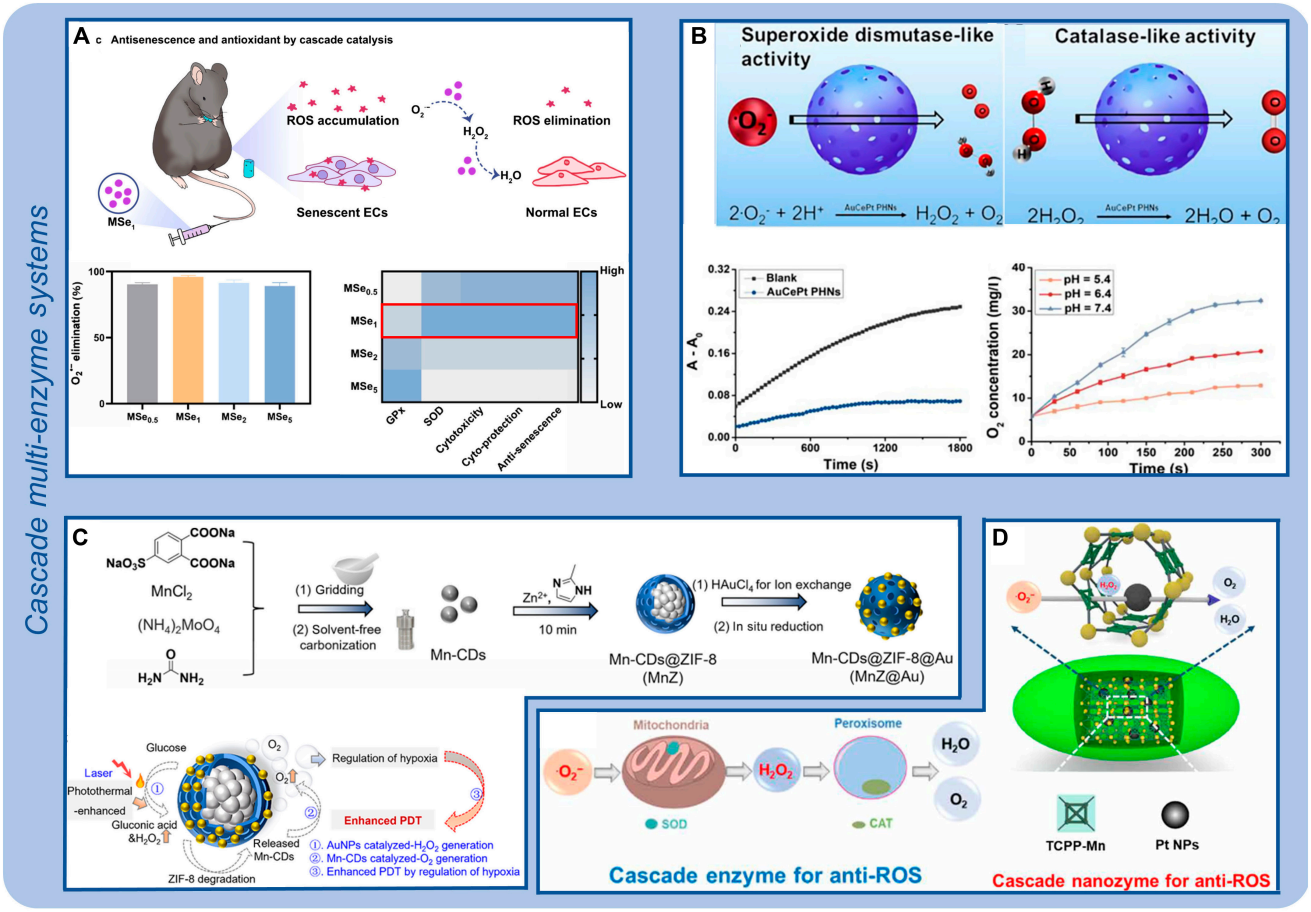
Cascade multi-enzyme systems. (A) MSe1 nanozymes with SOD- and GPx-like activities scavenge ROS in atherosclerotic plaques, slowing atherosclerosis in ApoE-deficient mice [140]. Copyright 2023, Wiley-VCH. (B) AuCePt nanozymes via SOD/CAT-like cascade catalysis scavenge ROS and deliver therapeutics, alleviating liver insulin resistance [137]. Copyright 2024, BioMed Central. (C) MnZ@Au cascade nanozymes with GOx- and CAT-like activities modulate tumor microenvironment, enhancing photodynamic therapy [141]. Copyright 2023, ACS. (D) Pt@PCN222-Mn nanozymes via SOD/CAT cascade catalysis clear ROS, exerting anti-inflammatory effects [136]. Copyright 2020, AAAS.

Besides switching that is turned on by a stimulus, cascade systems can also be made to handle ROS all the time. They do this through enzyme steps that work together. For CVD models, Liu and his team [140] reported MSe_1_ nanozymes. These combine SOD- and GPx-like activities, so they can clean up O_2_^•−^ and H_2_O_2_ one after another inside atherosclerotic plaques (Fig. 7A). Because this design maintains continuous ROS conversion, it reduces intermediate buildup, demonstrating the advantage of cascade catalysis that is coordinated in space. A similar idea is behind the AuCePt hollow multimetallic nanozymes made by Shen and colleagues [137]. In these nanozymes, many metal parts work together to help SOD- and CAT-like cascade reactions, and they also allow the delivery of therapeutic molecules (Fig. 7B). These examples show that having several metals work together and building them into one structure can keep cascade efficiency steady even in complex disease environments.

Cascade nanozyme design has also been used in disease settings where ROS control needs to be paired with changes in metabolism or the microenvironment. Luo and his group [141] built MnZ@Au cascade nanozymes. These nanozymes combine glucose oxidase (GOx)- and CAT-like activities (Fig. 7C). In this system, using up glucose creates reactive intermediates. Moreover, CAT-like activity makes oxygen, which helps with low oxygen levels. Together, this strategy enhances the therapeutic efficacy of ROS-based treatments and improves the spatial distribution of nanozymes via light-induced photothermal effects. For inflammatory diseases, Pt@PCN222-Mn and Pt@CND nanozymes use SOD/CAT cascade catalysis. This helps them reduce oxidative stress in an efficient way and also protect cell function (Fig. 7D) [136]. In these designs, cascade catalysis makes sure that ROS transformation is controlled rather than indiscriminate ROS scavenging or generation.

### Key environmental factors regulating antioxidant nanozyme activity

The biological application potential of antioxidant nanozymes stems not only from their intrinsic multi-enzyme mimetic catalytic activity but also from their adaptive regulatory properties to key physicochemical parameters in physiological and pathological microenvironments. pH, temperature, and substrate concentration, the 3 core factors that fluctuate dynamically in vivo, can precisely and reversibly tune the antioxidant catalytic behavior of nanozymes, endowing them with flexible functional adaptability that matches the complex in vivo environment [142].

pH has a basic and common control effect on the antioxidant catalytic cycle of nanozymes, and the activity trends are clear across all mainstream antioxidant systems [143]. For most antioxidant nanozymes, their SOD-like activity remains stable and effective in the neutral to weakly alkaline range, which fits perfectly with the physiological pH of healthy tissues [144]. CAT-like activity exhibits optimal activity at neutral pH, and at this pH, the core PCET process of the catalytic cycle is most efficient [145]. GPx-like activity also maintains high activity within the physiological pH range. Its efficiency depends a lot on the protonation state of the active center and the nucleophilic reactivity of the thiol group in glutathione [54]. The pH-dependent regulation arises from 3 main mechanisms. First, pH directly controls protonation and deprotonation of surface functional groups. This changes ROS substrate adsorption and electron transfer at the surface. Second, pH changes the valence balance and redox potential of variable-valence metal active centers, which then affects electron-donating and electron-accepting capability of catalytic sites. Third, pH also changes the activation energy of the slowest step in the catalytic cycle. This is especially true for reactions that need protons, like in CAT- and GPx-like catalysis [143]. Through rational structural design, antioxidant nanozymes can fine-tune their pH-responsive catalytic behavior. This lets them keep stable antioxidant function over a wider pH range and also allows selective activity regulation in different tissue microenvironments.

Temperature regulates the catalytic behavior of antioxidant nanozymes mainly by altering substrate mass transport rate, reaction rate constant, and activation energy barrier of each catalytic step following classic enzymatic kinetic principles [146]. Antioxidant nanozymes exhibit exceptional thermal stability, retaining more than 80% of their initial catalytic activity across a wide temperature range of 25 to 50 °C, in sharp contrast to natural antioxidant enzymes that undergo irreversible denaturation and loss of catalytic activity at temperatures above 40 °C [146]. This thermal stability ensures stable antioxidant function under physiological temperature fluctuations in vivo, as well as preserved catalytic activity after clinical high-temperature sterilization and long-term storage. For substrate concentration, the catalytic behavior of antioxidant nanozymes follows classic Michaelis–Menten kinetics, with catalytic efficiency showing a typical saturation kinetic characteristic with changes in substrate concentration [147]. Their antioxidant activity is tightly and reversibly tuned by the concentration of ROS substrates, including O_2_^•−^ and H_2_O_2_ , as well as the co-substrate glutathione. This concentration-dependent characteristic enables adaptive antioxidant regulation: Nanozymes rapidly clear ROS under high oxidative load in pathological lesions while maintaining minimal catalytic activity at physiological ROS levels to preserve normal endogenous redox signal transduction [148].

Therefore, antioxidant nanozymes exhibit highly tunable regulatory properties. They respond to pH, temperature, and substrate concentration. These properties are one of their main advantages over conventional antioxidant materials. Also, these adaptive traits let antioxidant nanozymes adapt to dynamic in vivo microenvironments. Then, they give a solid material foundation for their safe and efficient use in many biomedical settings.

## Biomedical and Therapeutic Applications of Antioxidant Nanozymes

Building on the rational design principles and performance optimization strategies detailed above, antioxidant nanozymes have evolved from catalytically active nanomaterials to versatile therapeutic and diagnostic platforms, addressing the core limitations of conventional antioxidant interventions in complex in vivo environments. Leveraging their tunable multienzyme-mimetic cascade activities, pathological microenvironment responsiveness, and flexible multifunctional integration, these nanozymes enable precise spatiotemporal regulation of ROS homeostasis across diverse disease settings. This section systematically summarizes the latest biomedical advances of antioxidant nanozymes, spanning neuroprotection, anti-inflammatory therapy, cardiovascular protection, tumor adjuvant therapy, regenerative medicine, and disease diagnosis, to delineate their translational value in oxidative stress-related disorders (Table 2).

**Table 2.** Summary of biomedical applications of antioxidant nanozymes

Application category	Core therapeutic strategy	Target disease models	References
Neuroprotection and neurodegenerative disease therapy	BBB penetration + SOD/CAT cascade ROS scavenging, inhibits neuroinflammation and neuronal death	Parkinson’s disease, Alzheimer’s disease, ischemic stroke, cerebral ischemia–reperfusion injury	[149–162]
Anti-inflammatory therapy	Lesion-targeted ROS/RNS clearance, modulates immune polarization, blocks oxidative stress-inflammation vicious cycle	Rheumatoid arthritis, ulcerative colitis, systemic sepsis, urinary tract infection	[163–169]
Cardiovascular protection	Plaque/cardiac-targeted ROS scavenging, regulates macrophage phenotype, improves vascular endothelial function	Atherosclerosis, myocardial ischemia–reperfusion injury, acute myocardial infarction	[170–175]
Tumor adjuvant therapy and normal tissue protection	Tumor microenvironment remodeling, radiotherapy/chemotherapy sensitization, normal tissue oxidative damage protection	Solid tumor radiosensitization, chemo/radiotherapy-induced organ injury	[176–181]
Tissue engineering and regenerative medicine	Scaffold-loaded sustained ROS scavenging, modulates inflammatory microenvironment, promotes stem cell differentiation	Diabetic chronic wounds, osteochondral regeneration, acute liver failure repair	[182–189]
Disease diagnosis	Antioxidant catalysis-mediated signal amplification, ROS scavenging improves in vivo sensing stability	Cardiac biomarker detection, in vivo neurotransmitter sensing, trace biomarker assay	[190–193]

### Neuroprotection and treatment of neurodegenerative diseases

Neurodegenerative diseases and central nervous system (CNS) injuries are characterized by persistent oxidative stress, excessive accumulation of ROS, mitochondrial dysfunction, and chronic neuroinflammation, together driving neuronal apoptosis, ferroptosis, and progressive functional loss. Excessive ROS directly inflicts oxidative damage on neuronal nucleic acids, membrane lipids, and functional proteins, further impairs the mitochondrial electron transport chain to amplify ROS production, and activates glial cells to establish a self-sustaining vicious cycle of oxidative stress and neuroinflammation in the pathological CNS microenvironment [149,150]. Conventional small-molecule antioxidants often fail to provide effective neuroprotection due to poor blood–brain barrier (BBB) penetration, rapid clearance, and limited ability to address the complex ROS spectrum present in the diseased brain [151]. In contrast, antioxidant nanozymes, by mimicking the cascade activities of natural enzymes such as SOD and CAT, can simultaneously scavenge multiple ROS species while offering tunable physicochemical properties for BBB penetration and lesion targeting, making them particularly suited for CNS therapy [152,153].

**Fig. 8.**
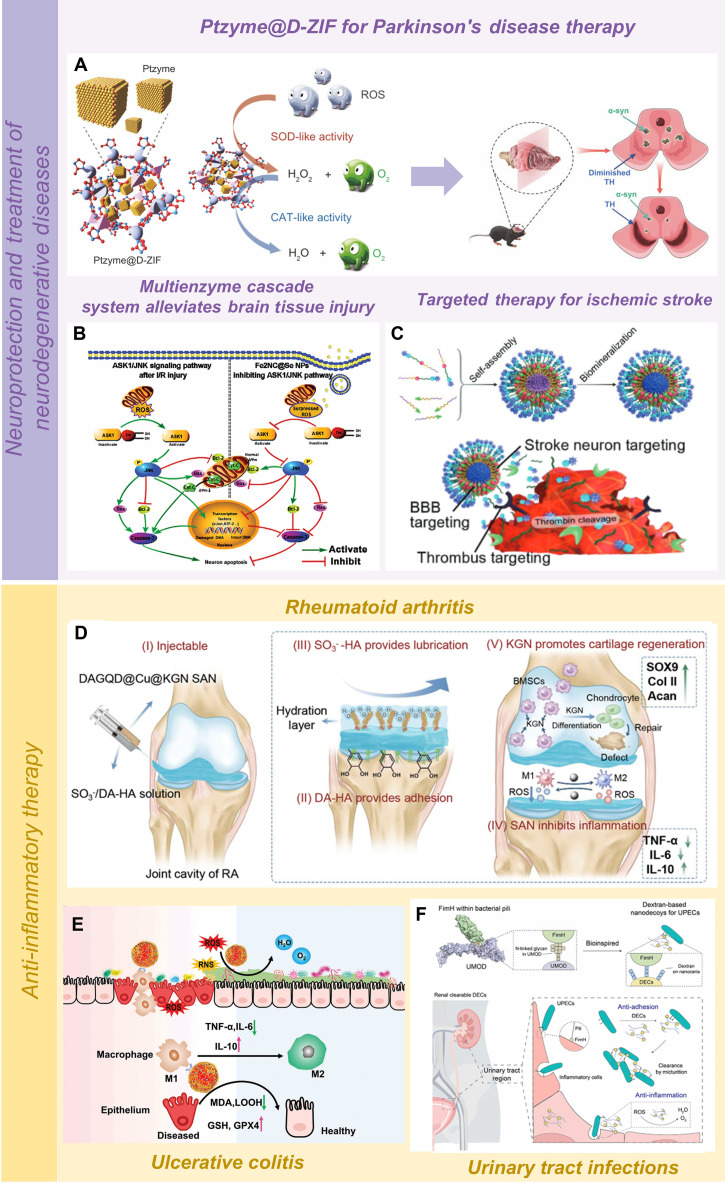
Neuroprotection, neurodegenerative disease treatment, and anti-inflammatory therapy. (A) Ptzyme@D-ZIF reduces brain ROS, suppresses neuronal cell death, and improves motor function in Parkinson’s disease models [155]. Copyright 2023, Springer Nature. (B) Fe_2_NC@Se scavenge RONS, inhibit apoptosis signaling, and alleviate cerebral ischemia–reperfusion injury [158]. Copyright 2022, Wiley-VCH. (C) PNzyme/MnO_2_ target thrombi, scavenge ROS, and exert thrombolytic-neuroprotective effects in ischemic stroke [160]. Copyright 2024, Wiley-VCH. (D) DAGQD@Cu@KGN SAN scavenge joint ROS, reduce inflammation, and aid cartilage repair for rheumatoid arthritis [165]. Copyright 2025, Springer Nature. (E) Se-CA nanozymes scavenge ROS/RNS and restore intestinal barrier function for ulcerative colitis [167]. Copyright 2025, Wiley-VCH. (F) Dextran-modified ceria nanozymes block bacterial adhesion, scavenge ROS, and treat urinary tract infection [169]. Copyright 2024, ACS.

In PD, oxidative stress is closely associated with dopaminergic neuron degeneration in the substantia nigra, where sustained ROS accumulation and neuroinflammation amplify neuronal vulnerability [154]. To address the dual challenges of BBB penetration and intracellular ROS clearance, a chiral MOF-encapsulated platinum nanozyme (Ptzyme@D-ZIF) was designed to exploit D-chirality-enhanced transport across the BBB (Fig. 8A) [155]. This structural design directly matches the need for efficient brain delivery, while the intrinsic SOD/CAT-like activity of Pt nanozymes enables continuous ROS scavenging in situ, thereby suppressing apoptosis and ferroptosis and improving motor function. Similarly, targeting mitochondrial oxidative stress, an early and central event in PD pathology, iron single-atom nanozymes delivered via mitochondrion-targeted liposomes (Mito@Fe-ISAzyme) were enriched in vulnerable brain regions using microneedle-assisted delivery [156]. This design aligns the catalytic site with the primary ROS source, enhancing antioxidant efficiency and mitigating neuroinflammation-driven neuronal damage.

Cerebral ischemia–reperfusion injury (CIRI) is characterized by a burst of reactive oxygen and nitrogen species (RONS) that occurs during reperfusion. This burst then overwhelms endogenous antioxidant defenses and also turns on apoptosis-related signaling pathways [157]. To address this acute and many-sided oxidative challenge, the team built dual-iron-atom nanozymes inside selenium-containing MOFs (Fe_2_NC@Se). This system works as a SOD/CAT/GPx-like cascade (Fig. 8B) [158]. Multiple redox-active centers are rationally integrated. This lets the nanozymes efficiently scavenge multiple RONS. Moreover, it also stops apoptosis signal-regulating kinase 1 (ASK1)/c-Jun N-terminal kinase (JNK)-mediated apoptosis. This shows that matching cascade catalytic ability to the reperfusion-related oxidative microenvironment is key for good neuroprotection.

In IS, oxidative stress is tightly coupled with thrombosis and inflammatory activation during reperfusion [159]. Thrombin-activated peptide-templated MnO_2_ nanozymes (PNzyme/MnO_2_) were therefore designed to respond specifically to the thrombotic microenvironment (Fig. 8C) [160]. This strategy enables site-specific thrombolysis while leveraging the intrinsic SOD- and CAT-like activities of MnO_2_ to scavenge ROS and suppress astrocyte activation and inflammatory cytokine release, demonstrating how coupling antioxidant catalysis with disease-triggered activation improves both efficacy and safety.

In Alzheimer’s disease (AD), chronic oxidative stress coexists with impaired antioxidant signaling and progressive neuronal dysfunction [161]. An MOF-based platform integrating targeting peptides, cerium oxide nanozymes, and a CRISPRa system (CMOPKP) was developed to cross the BBB and selectively accumulate in AD lesions [162]. The nanozyme moiety restores redox homeostasis via CAT-mediated ROS scavenging, while concurrent activation of the Nrf2 pathway reinforces endogenous antioxidant defense. This design strategy integrates sustained CAT catalytic activity to target the long-term redox dysregulation typical of neurodegenerative pathologies.

Collectively, the available studies indicate that the neuroprotective efficacy of antioxidant nanozymes does not solely derive from their high catalytic activity, but rather depends on the rational matching between the disease-specific oxidative microenvironment of the CNS and the tailored design features of nanozymes. These core design features include BBB permeability, subcellular targeting capability, cascade catalytic performance, and stimulus-responsive activation. Such design-driven strategies provide a rational framework for the development of nanozyme-based therapeutic systems targeting complex CNS disorders.

### Anti-inflammatory therapy

Autoimmune and infectious diseases often come with long-lasting inflammation and excessive ROS production. These 2 processes amplify each other and promote continuous tissue damage. Excessive ROS turns on the NF-κB and mitogen-activated protein kinase (MAPK) pro-inflammatory signaling pathways in both innate and adaptive immune cells. This also boosts the release of pro-inflammatory cytokines, damages tissue barriers through oxidative stress, and increases autoantigen exposure because of both infectious triggers and autoimmune responses. This leads to uncontrolled inflammatory cascades in the local pathological microenvironment. In these disease settings, ROS are not just byproducts of inflammation. They also actively control immune cell polarization, cytokine release, and barrier integrity. Conventional antioxidants or anti-inflammatory drugs often cannot stop this feedback loop because they have low stability or poor lesion-targeting ability or cannot simultaneously address oxidative stress and immune response dysregulation. Antioxidant nanozymes have adjustable redox activity and structural adaptability. Thus, they give us a practical way to match catalytic function with the inflammatory microenvironment. Then, they can remove local RONS and also adjust immune behavior [163].

Rheumatoid arthritis (RA) is characterized by a confined joint cavity environment with sustained oxidative stress, inflammatory macrophage infiltration, and progressive cartilage degeneration [164]. To address these coupled challenges, an injectable hydrogel system based on dopamine-modified hyaluronic acid (DA-HA) was designed to provide both mechanical lubrication and localized nanozyme delivery (Fig. 8D) [165]. Copper single-atom nanozymes (DAGQD@Cu SAN) embedded in the hydrogel mimic SOD/CAT activities, enabling continuous scavenging of O_2_^•−^, H_2_O_2_, and •OH directly within the joint space, where ROS concentrations are highest. This local antioxidant action suppresses M1 macrophage polarization and inflammatory cascades, while the sustained release of the cartilage repair factor KGN supports tissue regeneration. The design illustrates how matching antioxidant catalysis with joint-specific mechanical and inflammatory features enables effective, long-term RA management.

UC is a chronic inflammatory bowel disease whose inflammatory lesions are strictly restricted to the intestinal mucosa, with pathogenesis closely linked to macrophage-derived oxidative stress, epithelial cell ferroptosis, and gut microbiota dysbiosis [166]. To this end, arginine-modified selenium nanozymes (Se-CAs) have been rationally developed, which exploit the high expression of cationic amino acid transporter-2 (CAT2) on M1-type macrophages to achieve targeted accumulation at inflammatory sites (Fig. 8E) [167]. This targeted delivery strategy ensures that the antioxidant activity of the nanozymes is predominantly enriched at pathological lesions. Mechanistically, Se-CAs efficiently scavenge ROS/RNS and stabilize epithelial redox homeostasis, thereby protecting intestinal barrier integrity, down-regulating proinflammatory signaling, and restoring gut microbiota balance. This rational design exemplifies the precise matching between immune cell-targeted delivery and the spatial requirements of antioxidant therapy for localized inflammatory diseases.

By contrast, systemic sepsis is a life-threatening systemic inflammatory disorder characterized by diffuse whole-body oxidative stress, cytokine storm, and multiple organ dysfunction, rather than localized inflammatory lesions. For this pathological scenario, selenium nanoparticles coated with metal-polyphenol networks (denoted TZn@CSe) have been fabricated, which exhibit prolonged blood circulation, multi-modal antioxidant activity, and immunomodulatory capacity [168]. These nanozymes effectively reduce systemic ROS levels and promote the phenotypic switch of macrophages from proinflammatory M1 to regulatory phenotypes, thereby inhibiting excessive cytokine release and interrupting the vicious cycle between ROS overproduction and inflammatory amplification. This design highlights that, for systemic inflammatory diseases, the core design criteria for antioxidant nanozymes are excellent blood stability, biocompatibility, and immunomodulatory function, rather than lesion-specific retention.

For urinary tract infections (UTIs), the core pathological processes occur synchronously on the urinary mucosal surface, including bacterial adhesion, ROS-driven inflammatory injury, and urothelial barrier damage. To address this, dextran-modified ultrasmall ceria nanozymes (DECs) have been engineered, which bind to bacterial FimH proteins via multivalent interactions. This design not only blocks bacterial adhesion but also preserves the intrinsic SOD- and CAT-like catalytic activities of the nanozymes (Fig. 8F) [169]. Through sustained ROS scavenging, DECs alleviate inflammatory damage to the urothelium, promote mucosal tissue repair, and reduce the risk of recurrent infection. This bifunctional design integrating anti-adhesion and antioxidant activity demonstrates that antioxidant nanozymes can simultaneously block pathogen colonization and modulate host inflammatory responses.

Taken together, these studies show that effective anti-inflammatory nanozyme therapy depends on aligning catalytic behavior, targeting strategy, and material stability with the specific inflammatory microenvironment of each disease. Rather than acting solely as ROS scavengers, antioxidant nanozymes function as regulators of immune-redox coupling, enabling localized or systemic disruption of the ROS-inflammation feedback loop. This design-oriented perspective provides a practical framework for developing nanozyme-based interventions in complex autoimmune and infectious diseases.

### Cardiovascular protection

CVDs are still a top cause of death around the world. Their disease process is complex, so there are many challenges for treatment. In atherosclerosis (AS), 3 things happen together: oxidative stress in the endothelium, problems with lipid metabolism, and long-term inflammation. Together, they create a pathological environment. In that environment, ROS builds up, which makes plaques worse and disrupts the immune system off balance [170]. For myocardial ischemia/reperfusion injury (MIRI), the problem involves sudden bursts of ROS and a chain reaction of inflammation. This makes heart damage worse and also makes treatment harder [171]. All of this shows the need for antioxidant strategies designed specifically for the oxidative and inflammatory microenvironment of heart lesions. Antioxidant nanozymes are designed to match these disease features, with tunable catalytic activity, multi-enzyme mimetic function, and targeted delivery capability. These nanozymes can efficiently scavenge ROS and can also adapt to the lesion’s environment, thereby breaking the vicious cycle where oxidative stress and inflammation amplify each other, and helping maintain tissue stability [77,172].

**Fig. 9.**
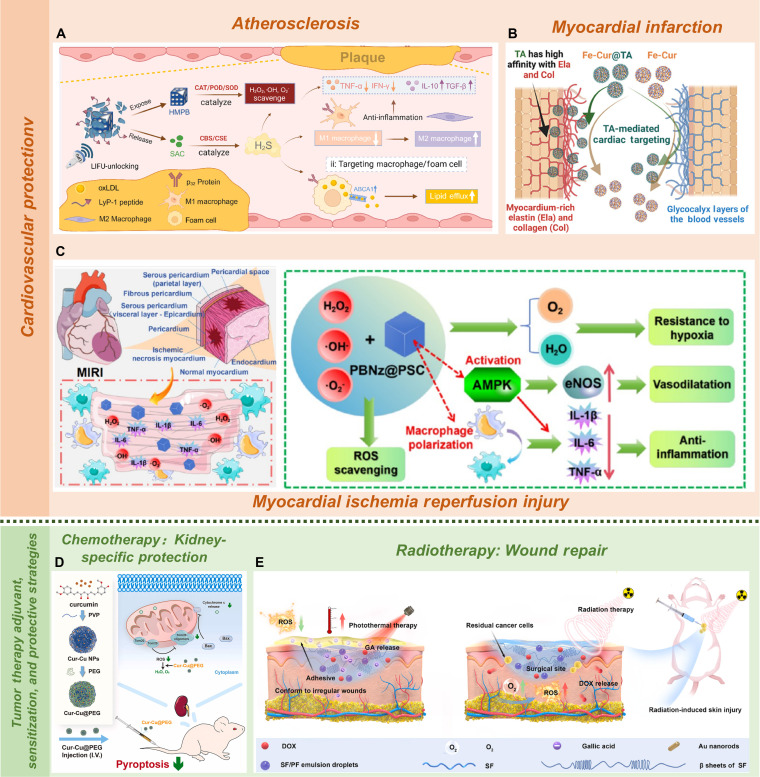
Roles of nanozymes in cardiovascular protection and tumor therapy adjuvants, sensitization, and protection. (A) LyP−1Lip@HS target plaque macrophages, scavenge ROS, and reverse atherosclerosis [173]. Copyright 2025, Elsevier. (B) Cardiac-targeting Fe-Cur@TA nanozymes scavenge free radicals and improve myocardial function [175]. Copyright 2024, Wiley-VCH. (C) PBNz@PSC modulate macrophages and aid myocardial repair [30]. Copyright 2025, ACS. (D) Curcumin–copper composite nanozymes scavenge cisplatin-induced ROS, protect kidneys, and alleviate acute renal injury [179]. Copyright 2025, Elsevier. (E) Photo-responsive antioxidant patches neutralize radiation-induced free radicals and promote skin wound repair [180]. Copyright 2024, Elsevier.

For treating AS, the design of nanozymes focuses on reaching the lesion and doing multiple jobs that match the local oxidative and inflammatory state. For instance, An and coworkers [173] made a liposome system with LyP-1 peptide on it (called LyP−1Lip@HS). This system carries Prussian blue nanozymes (HMPBs). It builds up mainly in plaque macrophages. Then, when ultrasound is applied, it releases natural H_2_S and also cleans up ROS using SOD-like activity. This design takes advantage of the high ROS level in plaques to push macrophages toward an anti-inflammatory M2 type. That helps promote cholesterol efflux and lowers plaque inflammation (Fig. 9A). Also, He and coworkers [174] created PBNZ@PP-Man nanozymes. These target both endothelial cells and macrophages. They get rid of ROS, stop monocytes from entering the tissue, and help clear out dying cells. All these design choices come directly from the disease features of AS. The nanozymes can fix the microenvironment balance and control inflammation very precisely.

For treating MIRI, nanozyme methods try to stop the inflammation–ROS cycle in the ischemic tissue. Liu and coworkers [175] made Fe-Cur@TA nanozymes with tannic acid on them. These nanozymes can target the heart, so they build up more in heart tissue (Fig. 9B). Their SOD-like catalytic activity neutralizes ROS. At the same time, they change the immune response by pushing M2 macrophages and lowering inflammatory cytokines. Long and coworkers [30] made polysaccharide-coated Prussian blue nanozymes (PBNz@PSCs). These have stronger SOD-like activity. They change macrophage types and help blood vessels work better by turning on adenosine monophosphate-activated protein kinase (AMPK) (Fig. 9C). Ye and coworkers [31] designed platinum single-atom nanozymes (PtsaN-C). These clean up ROS well using SOD/CAT-like activity. They also block the MAPK/JNK pathway. That keeps heart cells stable. In all these cases, the nanozyme design is guided by the specific ROS profile, immune condition, and tissue structure of the injured heart. That way, treatment is as precise as possible.

All these examples show that good heart nanozymes are not just simple ROS removers. Instead, they are carefully designed to match the oxidative and inflammatory microenvironment of each lesion. They combine targeted delivery, multiple enzyme activities, and immune system control to give wide-ranging protection. Such design-focused methods help blood vessel lining work better, control blood vessel tone, lower inflammation, and help tissue heal. This gives us a clear mechanism for precise treatment of complex CVDs.

### Tumor therapy adjuvant, sensitization, and protective strategies

In tumor tissues, the microenvironment is pathological, with hypoxia, high ROS, and lactate buildup. All these promote tumor growth and therapeutic resistance [176]. High ROS also impairs immune cells like T cells and dendritic cells, thereby reducing the efficacy of immunotherapy. This creates a demand for nanozymes that fit the tumor’s oxidative and metabolic state, with the ability to regulate ROS, modulate metabolism, and support antitumor immunity.

Antioxidant nanozymes are rationally designed to directly target these pathological hallmarks. For example, CoMnFe layered double oxide (LDO) nanozymes possess a 2D structure, which provides a large specific surface area and abundant catalytic active sites, while the doped transition metals facilitate rapid electron transfer. These nanozymes exhibit CAT-, POD-, and OXD-like activities, thereby enabling multiple synergistic functions: converting H_2_O_2_ into O_2_ to alleviate tumor hypoxia, generating toxic ROS to sensitize radiotherapy, and scavenging lactate to inhibit tumor tissue repair and metabolic reprogramming [177]. Benefiting from the precise matching between the structural and functional features of LDO nanozymes and the ROS- and lactate-enriched tumor microenvironment, these nanozymes achieve simultaneous multi-pathway regulation, which substantially enhances the radiotherapy efficacy of uveal melanoma and holds promising application potential for other hypoxia-sensitive tumors.

Antioxidant nanozymes also protect normal tissues from damage caused by treatment. Radiotherapy and chemotherapy often cause severe damage to the kidneys, heart, and skin because of too much ROS [178]. For example, curcumin–copper nanozymes act like SOD. They remove ROS made by cisplatin therapy. This alleviates cisplatin-induced nephrotoxicity and blocks caspase-3-mediated apoptosis (Fig. 9D) [179]. Also, photoresponsive patches with gallic acid and gold nanorods neutralize free radicals and help heal skin wounds from radiation (Fig. 9E) [180]. These examples show that nanozyme design can be changed to match tumor features or to fix off-target oxidative stress. This gives tissue-specific protection.

The tumor immune microenvironment is also a key therapeutic target. Excessive ROS accumulation in tumors impairs the effector function of T cells and dendritic cells, thereby attenuating antitumor immunity [176]. In contrast, antioxidant nanozymes maintain intracellular redox homeostasis, thus protecting immune effector cells and exerting synergistic effects with immunotherapy. This strategy achieves bidirectional regulation: It enhances the antitumor efficacy of treatment while protecting normal tissues and preserving systemic immune function [181].

In short, tumor-targeted antioxidant nanozymes are rationally designed to match the oxidative and metabolic characteristics of the tumor microenvironment, enabling them to exert multiple synergistic functions including ROS scavenging, lactate clearance, hypoxia alleviation, and immunomodulation. This design-driven strategy significantly improves antitumor therapeutic outcomes while protecting normal tissues, endowing these nanozymes with important application value in comprehensive antitumor therapeutic regimens.

### Tissue engineering and regenerative medicine

Because of their tunable biomimetic catalytic activity, antioxidant nanozymes have emerged as promising functional components in tissue engineering and regenerative medicine [182]. In acutely injured tissues, excess ROS directly disrupts the extracellular matrix integrity, activates pro-apoptotic and pro-inflammatory signaling cascades, and also severely impairs endogenous stem cell function. This further hinders the normal tissue repair processes [93]. Upon the incorporation of nanozymes into biomaterial scaffolds, localized and sustained ROS elimination can be achieved, alongside concurrent remodeling of the inflammatory microenvironment. This dual function keeps implanted or endogenous cells alive, helps them move from proliferation to differentiation, and also supports the shift from the inflammatory phase to the remodeling phase. All these together lead to efficient tissue reconstruction. Notably, mechanical stimulation and cellular mechano-transduction are now recognized as core regulators of tissue development, regeneration, and remodeling. Recent work also shows that the biomechanical properties of biomaterial scaffolds can work together with antioxidant nanozymes. This gives multi-dimensional control over the regenerative microenvironment [183]. The stiffness, viscoelasticity, and topological structure of scaffolds can precisely change cell fate and tissue repair progression through mechano-transduction pathways. At the same time, nanozymes take care of oxidative and inflammatory barriers in the lesion site. Together, they push forward efficient tissue repair and functional reconstruction [183].

**Fig. 10.**
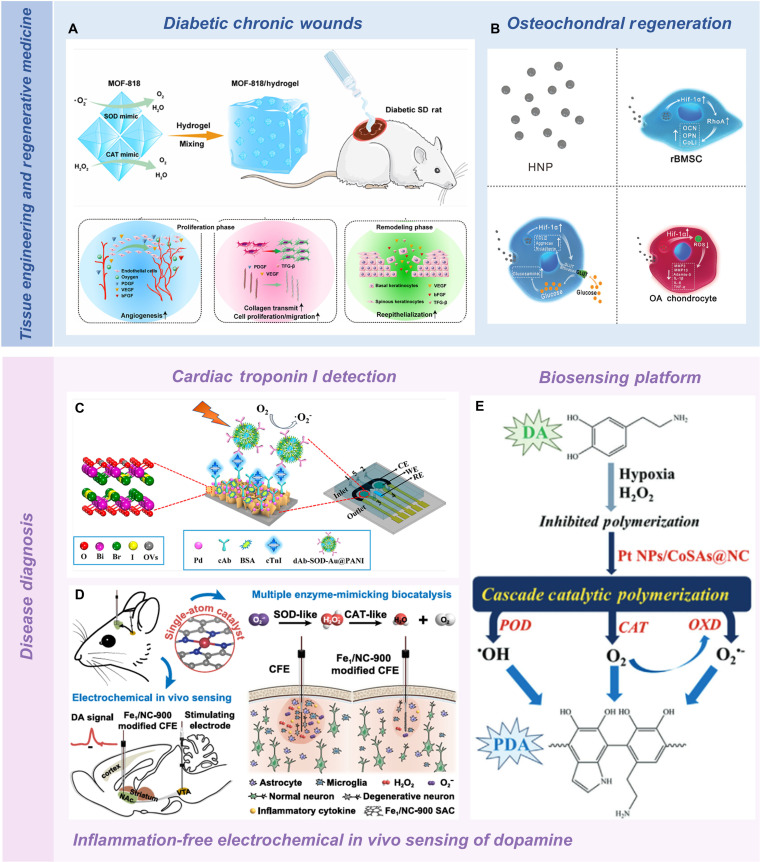
Nanozymes for tissue engineering, regenerative medicine, and disease diagnosis. (A) MOF-818 nanozymes in thermo-responsive hydrogels clear ROS, reduce oxidative damage, and accelerate chronic diabetic wound healing [184]. Copyright 2022, ACS. (B) Antioxidant nanoparticles in 3D-printed scaffolds neutralize joint ROS, protect chondrocytes, and promote osteochondral regeneration [188]. Copyright 2022, Wiley-VCH. (C) The Au@PANI-based microfluidic sensor uses an oxygen cycle cascade to enhance signal and detect cardiac troponin I ultrasensitively [192]. Copyright 2021, ACS. (D) FeN_4_ single-atom catalysts scavenge ROS, suppress neuroinflammation, and improve long-term reliability of in vivo dopamine detection [193]. Copyright 2024, Springer Nature. (E) Pt NPs/CoSAs@NC nanozymes enable femtomolar-level detection of PTP1B via cascade-catalyzed signal amplification [138]. Copyright 2024, Wiley-VCH.

In chronic diabetic wounds, for example, MOF-818 nanozymes incorporated into thermoresponsive hydrogels address the persistent oxidative microenvironment by mimicking SOD/CAT-like activities, achieving prolonged local ROS clearance. This targeted antioxidant function not only reduces oxidative damage to surrounding cells but also promotes resolution of inflammation, enabling healing outcomes comparable to daily clinical use of epidermal growth factor gel with a single application, thus providing an efficient approach for otherwise hard-to-heal wounds (Fig. 10A) [184]. In the same diabetic wound repair scenario, a glucose-responsive self-switchable Zn–Fe MOF nanozyme-hydrogel system has been developed to address the coupled hyperglycemia, bacterial infection, and oxidative stress barriers. This system integrates GOx and insulin to realize cascade catalysis: Hyperglycemia triggers its peroxidase-like activity for antibacterial hydroxyl radical generation and on-demand insulin release, with catalytic activity self-terminated under normoglycemia to prevent hypoglycemia, ultimately accelerating wound healing [185]. Likewise, in solar dermatitis, an acute UVB-induced skin radiation injury driven by excessive ROS accumulation and inflammatory cascade, a π-conjugated polyphthalocyanine-based artificial antioxidase with highly surface-exposed Ru active sites (HSE-PPcRu) has been developed. With robust SOD/CAT-like ROS-scavenging activity, this system effectively neutralizes excess ROS, modulates MAPK/NF-κB inflammatory pathways, inhibits keratinocyte damage, and ultimately alleviates UVB-induced solar dermatitis in vivo [186].

Matching the scaffold stiffness to that of native bone or cartilage further enhances the pro-regenerative effect. Biomimetic matrix stiffness promotes the osteogenic or chondrogenic differentiation of mesenchymal stem cells via mechano-transduction signaling, which acts synergistically with the antioxidant activity of nanozymes [183]. Zinc-based biodegradable materials have been extensively studied for bone scaffolds due to their moderate degradation rate and good biocompatibility. In addition, zinc-based nanozymes can scavenge ROS in bone defects, thereby reducing oxidative stress. This improves the bone healing efficacy of zinc scaffold systems [187]. For bone and cartilage repair, the intra-articular environment has excessive oxidative stress and impaired cell growth. Incorporation of antioxidant nanozymes into 3D-printed wollastonite bioceramic scaffolds enables the system to scavenge excess ROS via SOD-like activity. This protects chondrocytes and enhances glucose metabolism, supporting chondrocyte growth and maturation. The nanozymes also promote the osteogenic differentiation of bone marrow-derived mesenchymal stem cells, leading to better osteochondral integration than pristine scaffolds alone (Fig. 10B) [188].

For acute liver failure, excessive ROS and inflammatory storms in the liver impair hepatocyte survival and tissue regeneration. Antioxidative gold nanoclusters (NAC-Au NCs) with SOD/CAT-like activity are incorporated into 3D-printed liver sinusoid scaffolds, which are combined with hydrogels carrying hepatocyte-like cell spheroids (HLCs). This construct provides physical support and reduces ROS levels. As a result, oxidative stress is alleviated, inflammation is suppressed, and liver function indices are improved, facilitating liver tissue repair and functional recovery [189]. Together, these examples illustrate a general design principle: By matching the catalytic properties of nanozymes to the specific oxidative and inflammatory characteristics of a tissue microenvironment, alongside the rational design of scaffold biomechanical properties to recapitulate native tissue mechanical cues, it is possible to achieve multilevel, synergistic interventions. Such strategies not only protect resident and implanted cells from ROS-induced damage but also actively modulate the local biochemical and biomechanical milieu to enhance angiogenesis, cell migration, stem cell differentiation, and tissue regeneration, providing a robust framework for developing high-efficiency, intelligent regenerative platforms.

### Disease diagnosis

Antioxidant nanozymes exhibit catalytic functions analogous to natural antioxidant enzymes, enabling precise regulation of biological catalytic signals. This further enhances the sensitivity and selectivity of biomarker detection through cascade signal amplification, substrate-specific redox catalysis, and microenvironment-responsive activity regulation [190,191]. Researchers also use atomic-level engineering to optimize the surface coordination environment, morphology, and composite material interfaces of nanozymes, which significantly improves their catalytic rate and substrate specificity. Such designs achieve 2 core functions: First, they remove ROS efficiently; second, they turn antioxidant processes into measurable electrochemical or photoelectrical signals. The catalytic activity directly contributes to signal amplification and sensing performance. This method exhibits excellent performance in complex biological samples and is especially effective at reducing biointerface interference and improving signal stability and reliability.

For example, Feng et al. made a dual-channel microfluidic sensor based on SOD-Au@PANI. In this sensor, SOD breaks down O_2_^•−^ into H_2_O_2_ and O_2_. Subsequently, Au@PANI breaks down H_2_O_2_ to make O_2_ again. The regenerated O_2_ acts as an electron acceptor, thereby enhancing the cathodic photocurrent. Benefiting from this mechanism, the sensor can detect cardiac troponin I (cTnI) with very high sensitivity with a limit of detection of 0.042 pg mL^−1^. There is an oxygen cycle cascade. This cascade changes the antioxidant process into positive-feedback amplified photoelectric signal. As a result, trace biomarkers can be recognized reliably (Fig. 10C) [192]. For sensing in vivo, Gao et al. designed a FeN_4_ single-atom nanozyme with dual antioxidant activity (SOD/CAT-like) and electrocatalytic activity toward dopamine oxidation. After being implanted into rat brains, the nanozymes eliminate interfacial ROS, alleviate neuroinflammation, and achieve long-term stable dopamine detection. This design fixes the signal errors caused by inflammation in traditional electrochemical sensors (Fig. 10D) [193].

In addition, Wang et al. fabricated a Pt NP/CoSA@NC multifunctional nanozyme. It has POD-, OXD-, and CAT-like activities. These nanozymes help dopamine polymerize in situ into polydopamine (PDA). The generated PDA acts as a photoelectric polarity regulator, switching the cathodic current of ZnCdS photoanode to anodic current. The polymerization causes signal amplification. This allows detection of protein tyrosine phosphatase 1B (PTP1B) at femtomolar levels, with a detection limit of 0.04 fM (Fig. 10E) [138]. This method uses antioxidant catalytic activity to enhance detection sensitivity and adopts signal aggregation to improve anti-interference performance, exhibiting good reliability and specificity for trace biomarker detection.

In conclusion, antioxidant nanozymes function as efficient signal amplifiers in disease diagnosis and can cooperate with diverse signal transduction pathways to accomplish high-sensitivity and high-specificity biological detection. By carefully designing catalytic functions and material interfaces, these systems can reduce interference in complex biological samples, promoting the clinical transformation of biomarker detection technology. The main advantage is the deep integration of catalytic activity and signal conversion. This offers a new way for early diagnosis and precise monitoring.

## Challenges and Future Perspectives

Although antioxidant nanozymes have achieved substantial progress in basic research and practical applications for disease therapy, tissue regeneration, and precision medicine, translating these bench-synthesized materials into clinical practice still faces numerous obstacles. These limitations derive from 2 major aspects: inherent drawbacks of nanozyme materials themselves, as well as the complexity of biological microenvironments and actual clinical therapeutic demands. First, nanozymes are required to maintain high catalytic activity and selectivity, and accurately distinguish physiological ROS from pathological ROS, so as to avoid disturbing normal cellular signal transduction. Second, the in vivo behaviors of nanozymes are extremely intricate. The interactions between nanozymes, biological macromolecules, and the immune system remain incompletely elucidated, which directly determines their in vivo therapeutic efficacy and biological safety. Furthermore, different laboratories use different methods and standards to test these nanozymes. This results in poor comparability of research findings across independent studies. Additional critical bottlenecks restricting clinical translation include scalable production, cost control, and efficient in vivo delivery of nanozyme systems. More critically, the requirements for redox homeostasis regulation vary drastically across different disease contexts and tissue types, which necessitates the development of stimulus-responsive nanozymes with selective ROS modulation capacity. Specifically, these nanozymes should selectively scavenge pathological ROS associated with disease progression while preserving physiological ROS that are essential for normal biological functions. Collectively, these interconnected challenges highlight the urgent need for a systematic, full-chain strategy spanning rational material design, optimized in vivo delivery, and standardized clinical translation. Herein, we systematically summarize 8 core unresolved challenges currently facing the field.

### Enhancing activity and selectivity

A core challenge for antioxidant nanozymes is that their catalytic efficiency and specificity toward particular ROS are generally lower than those of natural enzymes. Current strategies aim to optimize composition, size, morphology, and surface chemistry. However, these approaches cannot fully resolve critical issues: Nanozymes often struggle to distinguish between physiological ROS and pathological excess. While scavenging harmful radicals, they may inadvertently affect signaling molecules, and some nanozymes generate secondary reactive species, such as hydroxyl radicals, during catalysis. These dual limitations in activity and selectivity severely restrict therapeutic precision in complex biological environments, forming a primary bottleneck for clinical translation. Emerging approaches include biomimetic interface design and ML-assisted optimization; however, achieving the specificity of natural enzymes remains challenging.

### Complexity of catalytic mechanisms

The in vivo catalytic mechanisms of nanozymes remain poorly clarified. Most studies are done in vitro with simple setups, and these setups do not capture the real complexity of blood, interstitial fluid, or tissue microenvironments. Once entering organisms, nanozymes are rapidly coated by various biomolecules to form dynamic protein coronas. This corona can hide or change the catalytic sites. When nanozymes meet immune parts or cell receptors, unwanted biological effects may happen, and these could alter metabolic pathways. Because we know so little about how the whole process works, it is hard to guess how well they will catalyze at disease sites. It is also tough to tell if they might disturb normal body functions. Advances in in situ characterization and theoretical calculations have been made, but real-time monitoring of nanozyme behavior in vivo is still very hard to do. This makes smart design difficult.

### Standardization

A pressing issue is the lack of a unified evaluation system. Globally, more than 20 methods are used to measure nanozyme activity. For example, SOD-like activity alone has 5 major assay techniques, each with different parameters (temperature, pH, and substrate concentration), leading to poor comparability. Instrumentation choices for characterization (e.g., microscopy and spectroscopy) further influence the interpretation of structure–activity relationships. Reference standards vary across studies, complicating cross-comparison. Without standardization, the literature data are inconsistent, and cross-laboratory performance assessments lack meaning. Establishing a complete standard system covering activity assays, toxicity evaluation, and in vitro–in vivo correlation remains a long-term goal.

### Biocompatibility and safety

Long-term safety is a key requirement for clinical translation. Most current studies only focus on short-term in vitro cytotoxicity detection, while the full in vivo transport pathways and metabolic fate of nanozymes have not been systematically clarified. Animal tests show that some nanozymes build up in the liver and spleen at levels reaching 60% of the given dose, but there is very little information about what happens to them after 3 months. Metal-based nanozymes can shed ions when they are inside acidic lysosomes, and this release can lead to damage in mitochondria and cause DNA oxidation. Some carbon-based nanozymes may switch on the NLRP3 inflammasome, a process that can then set off chronic fibrosis [194]. Because of these dangers, a well-planned safety assessment framework is needed. Such a framework must include studies on immunogenicity, reproductive toxicity, neurotoxicity, and also an evaluation of how much breakdown products accumulate in tissues. At the moment, there are no standardized safety guidelines made just for nanozymes.

### Scale-up production and cost

Moving from laboratory-scale synthesis to industrial production is a key hurdle. High-performance nanozymes typically need precious metal precursors, high-temperature pyrolysis, or vacuum deposition. These methods give only gram amounts and are expensive. As an example, platinum-based nanozymes cost can be substantially more expensive than natural enzymes, depending on synthesis and purification processes. It is hard to scale up and still keep uniform particle properties. Microfluidic approaches make uniform particles but have low throughput. Conventional hydrothermal methods give wider size distributions and batch variation over 30%. Continuous-flow reactors and biomimetic mineralization are being explored as potential solutions. But making kilogram-scale batches with consistent single-particle performance at clinically acceptable cost is still a big challenge.

### Targeted delivery efficiency

Efficient and precise delivery to disease sites (ischemia/reperfusion zones, inflammatory lesions, or tumor microenvironments) is essential for therapeutic efficacy. Strategies include surface functionalization with antibodies, peptides, or aptamers and the exploitation of physicochemical properties such as magnetic or pH responsiveness. Nevertheless, physiological barriers, reticuloendothelial clearance, the vascular endothelium, and high tumor interstitial pressure limit effective accumulation. Improving delivery efficiency requires smarter, biomimetic systems and a deeper understanding of in vivo transport mechanisms.

### Clinical translation barriers

The pathway from the laboratory to the clinic faces numerous challenges. Preclinical animal models cannot fully mimic the complexity of human diseases (e.g., murine liver metabolism is about 7 times faster than that in humans, with distinct oxidative stress pathways). Regulatory requirements necessitate Good Laboratory Practice (GLP)-compliant studies on acute, chronic, and genotoxic toxicity, which often involve costs in the millions. Nanozyme-specific pharmacokinetics and immunotoxicity studies currently lack clear regulatory guidance, extending development timelines by several years. Industrial-scale production requires Good Manufacturing Practice (GMP)-compliant facilities, yet it remains uncertain whether nanozymes can maintain their catalytic activity and structural integrity under standard sterilization or other stringent industrial conditions. To date, few antioxidant nanozymes have advanced to phase II clinical trials, with only a handful of candidates entering early-stage clinical development—most notably CNM-Au8, an antioxidant catalytic gold nanozyme developed as a potential therapeutic for ALS. Overcoming these barriers will require coordinated efforts across academia, industry, and clinical practice, as well as tailored regulatory strategies.

### Maintaining redox homeostasis

The ultimate challenge is the precise regulation of redox balance. Physiological ROS are key signaling molecules in growth, differentiation, and immunity. Most existing nanozymes are engineered to maintain high and sustained activity. While scavenging pathological ROS, they may interfere with physiological signaling and thereby induce rebound effects. Recent advances have led to the development of ROS concentration-dependent dynamic responsive nanozymes, whose antioxidant catalytic activity can be adaptively tuned in response to local ROS levels, thus effectively preserving the normal signaling function of physiological ROS while eliminating excess pathological ROS [195]. Different diseases require context-specific modulation: Tumors may require local ROS elevation, whereas neurodegenerative diseases benefit from gentle, sustained ROS control. Ideal nanozymes would possess biosensing capability to dynamically adjust activity according to local ROS levels. Although pH-responsive, light-controlled, and such ROS-responsive dynamic nanozymes have been explored, achieving truly intelligent, on-demand antioxidant function remains challenging, requiring innovations in both material design and precise in vivo control.

In summary, this review systematically sorts out the definition, rational design, and catalytic optimization of antioxidant nanozymes. There is a growing consensus that building nanozymes based on the structural and functional traits of natural enzymes offers a clear path toward high catalytic efficiency and selectivity. Nature has fine-tuned its enzymatic systems across billions of years of evolution, and deep catalytic insights are embedded in atomic arrangements and the regulation of microenvironments. So, as these principles are uncovered and applied to material design, nanozyme research is moving from trial-and-error work toward more mechanism-guided engineering. Also, bioinspired antioxidant nanozymes are expected to become a central focus in both catalysis and biomedical studies. Finally, the aim of this review is to spark wider interest, offer conceptual ideas, and support the rational development of next-generation nanozymes that can achieve precise and controllable redox regulation in living systems.

## References

[1] Correia AS, Cardoso A, Vale N. Oxidative stress in depression: The link with the stress response, neuroinflammation, serotonin, neurogenesis and synaptic plasticity. Antioxidants. 2023;12(2):470. 36830028 10.3390/antiox12020470 PMC9951986

[2] Liang X, Weng J, You Z, Wang Y, Wen J, Xia Z, Huang S, Luo P, Cheng Q. Oxidative stress in cancer: From tumor and microenvironment remodeling to therapeutic frontiers. Mol Cancer. 2025;24(1):219. 40847302 10.1186/s12943-025-02375-x PMC12372290

[3] Xiao L, Gu C, Xiang Y. Orthogonal activation of RNA-cleaving DNAzymes in live cells by reactive oxygen species. Angew Chem Int Ed Engl. 2019;58(40):14167–14172. 31314942 10.1002/anie.201908105

[4] Wen P, Sun Z, Gou F, Wang J, Fan Q, Zhao D, Yang L. Oxidative stress and mitochondrial impairment: Key drivers in neurodegenerative disorders. Ageing Res Rev. 2025;104: Article 102667. 39848408 10.1016/j.arr.2025.102667

[5] Korovila I, Hugo M, Castro José P, Weber D, Höhn A, Grune T, Jung T. Proteostasis, oxidative stress and aging. Redox Biol. 2017;13:550–567. 28763764 10.1016/j.redox.2017.07.008 PMC5536880

[6] Vaziri ND, Dicus M, Ho ND, Boroujerdi-Rad L, Sindhu RK. Oxidative stress and dysregulation of superoxide dismutase and NADPH oxidase in renal insufficiency. Kidney Int. 2003;63(1):179–185. 12472781 10.1046/j.1523-1755.2003.00702.x

[7] Kinnula VL, Crapo JD. Superoxide dismutases in the lung and human lung diseases. Am J Respir Crit Care Med. 2003;167(12):1600–1619. 12796054 10.1164/rccm.200212-1479SO

[8] Calabrese EJ, Canada AT. Catalase: Its role in xenobiotic detoxification. Pharmacol Ther. 1989;44(2):297–307. 2519346 10.1016/0163-7258(89)90069-7

[9] Canli Ö, Alankuş YB, Grootjans S, Vegi N, Hültner L, Hoppe PS, Schroeder T, Vandenabeele P, Bornkamm GW, Greten FR. Glutathione peroxidase 4 prevents necroptosis in mouse erythroid precursors. Blood. 2016;127(1):139–148. 26463424 10.1182/blood-2015-06-654194 PMC4705604

[10] Uematsu T, Nagashima S, Umemura K, Kanamaru M, Nakashima M. Pharmacokinetics and safety of intravenous recombinant human superoxide dismutase (NK341) in healthy subjects. Int J Clin Pharmacol Ther. 1994;32(12):638–641. 7881700

[11] Ashrafi AM, Bytesnikova Z, Barek J, Richtera L, Adam V. A critical comparison of natural enzymes and nanozymes in biosensing and bioassays. Biosens Bioelectron. 2021;192: Article 113494. 34303137 10.1016/j.bios.2021.113494

[12] Krusic PJ, Wasserman E, Keizer PN, Morton JR, Preston KF. Radical reactions of C_60_. Science. 1991;254(5035):1183–1185. 17776407 10.1126/science.254.5035.1183

[13] Gao L, Zhuang J, Nie L, Zhang J, Zhang Y, Gu N, Wang T, Feng J, Yang D, Perrett S, et al. Intrinsic peroxidase-like activity of ferromagnetic nanoparticles. Nat Nanotechnol. 2007;2(9):577–583. 18654371 10.1038/nnano.2007.260

[14] Wei H, Wang E. Nanomaterials with enzyme-like characteristics (nanozymes): Next-generation artificial enzymes. Chem Soc Rev. 2013;42(14):6060–6093. 23740388 10.1039/c3cs35486e

[15] Ma L, Liang Z, Hou Y, Zhang R, Fan K, Yan X. Nanozymes and their potential roles in the origin of life. Adv Mater. 2025;37(6):2412211. 10.1002/adma.202412211 39723709

[16] Zhang R, Yan X, Gao L, Fan K. Nanozymes expanding the boundaries of biocatalysis. Nat Commun. 2025;16(1):6817. 40707464 10.1038/s41467-025-62063-8 PMC12290111

[17] Ma Y, Pan J, Ju C, Yu X, Wang Y, Li R, Hu H, Wang X, Hao D. Antioxidant nanozymes: Current status and future perspectives in spinal cord injury treatments. Theranostics. 2025;15(13):6146–6183. 40521206 10.7150/thno.114836 PMC12159832

[18] Xue B, Lu Y, Wang S, Xiao Q, Luo X, Wang Y, Yan X, Yang Z, Jiang B. The emerging role of nanozymes in ocular antioxidant therapy. Nano Today. 2024;58: Article 102448.

[19] Zhao J, Guo F, Hou L, Zhao Y, Sun P. Electron transfer-based antioxidant nanozymes: Emerging therapeutics for inflammatory diseases. J Control Release. 2023;355:273–291. 36731800 10.1016/j.jconrel.2023.01.068

[20] Zhou S, Cai H, He X, Tang Z, Lu S. Enzyme-mimetic antioxidant nanomaterials for ROS scavenging: Design, classification, and biological applications. Coord Chem Rev. 2024;500: Article 215536.

[21] Tian R, Xu J, Luo Q, Hou C, Liu J. Rational design and biological application of antioxidant nanozymes. Front Chem. 2021;8: Article 2020. 10.3389/fchem.2020.00831 PMC7905316 33644000

[22] Liu Y, Xiao Z, Chen F, Yue L, Zou H, Lyu J, Wang Z. Metallic oxide nanomaterials act as antioxidant nanozymes in higher plants: Trends, meta-analysis, and prospect. Sci Total Environ. 2021;780: Article 146578. 34030327 10.1016/j.scitotenv.2021.146578

[23] He Q, Zhang L. Design of carbon dots as nanozymes to mediate redox biological processes. J Mater Chem B. 2023;11(23):5071–5082. 37219483 10.1039/d2tb02259a

[24] Vallabani NVS, Karakoti AS, Singh S. ATP-mediated intrinsic peroxidase-like activity of Fe3O4-based nanozyme: One step detection of blood glucose at physiological pH. Colloids Surf B Biointerfaces. 2017;153:52–60. 28214671 10.1016/j.colsurfb.2017.02.004

[25] Shah J, Purohit R, Singh R, Karakoti AS, Singh S. ATP-enhanced peroxidase-like activity of gold nanoparticles. J Colloid Interface Sci. 2015;456:100–107. 26111515 10.1016/j.jcis.2015.06.015

[26] Ma L, Zheng J-J, Zhou N, Zhang R, Fang L, Yang Y, Gao X, Chen C, Yan X, Fan K. A natural biogenic nanozyme for scavenging superoxide radicals. Nat Commun. 2024;15(1):233. 38172125 10.1038/s41467-023-44463-w PMC10764798

[27] Yang X, Tan M, Guo J, Xiang J, Yin F, Deng J, Luo J, Xiao S, Mo M, Wang H, et al. PdZn/CoSA-NC nanozymes with highly efficient SOD/CAT activities for treatment of osteoarthritis via regulating immune microenvironment. Adv Funct Mater. 2024;34(41):2401963.

[28] Wang L, Yu S, Wang J, Wang Q, Mao Y, Zheng L. Manganese-doped carbon nanospheres with robust peroxidase-like activity for the colorimetric detection of total antioxidant capacity. Food Chem. 2025;484: Article 144349. 40252442 10.1016/j.foodchem.2025.144349

[29] Rzigalinski BA, Carfagna CS, Ehrich M. Cerium oxide nanoparticles in neuroprotection and considerations for efficacy and safety. Wiley Interdiscip Rev Nanomed Nanobiotechnol. 2017;9(4): Article e1444. 10.1002/wnan.1444 PMC5422143 27860449

[30] Long M, Wang L, Kang L, Liu D, Long T, Ding H, Duan Y, He H, Xu B, Gu N. Prussian blue nanozyme featuring enhanced superoxide dismutase-like activity for myocardial ischemia reperfusion injury treatment. ACS Nano. 2025;19(4):4561–4581. 39835774 10.1021/acsnano.4c14445

[31] Ye T, Chen C, Wang D, Huang C, Yan Z, Chen Y, Jin X, Wang X, Ding X, Shen C. Protective effects of Pt-N-C single-atom nanozymes against myocardial ischemia-reperfusion injury. Nat Commun. 2024;15(1):1682. 38396113 10.1038/s41467-024-45927-3 PMC10891101

[32] Liang H, Xian Y, Wang X. Preparation and application of single-atom nanozymes in oncology: A review. Front Chem. 2024;12:1442689. 39189019 10.3389/fchem.2024.1442689 PMC11345252

[33] Chen Z, Yu Y, Gao Y, Zhu Z. Rational design strategies for nanozymes. ACS Nano. 2023;17(14):13062–13080. 37399457 10.1021/acsnano.3c04378

[34] Yu Y, Zhang M, Fan K. Artificial intelligence-driven revolution in nanozyme design: From serendipity to rational engineering. Mater Horiz. 2025;12(19):7779–7813. 40569573 10.1039/d5mh00719d

[35] Abedi N, Sajadi-Javan ZS, Kouhi M, Ansari L, Khademi A, Ramakrishna S. Antioxidant materials in oral and maxillofacial tissue regeneration: A narrative review of the literature. Antioxidants. 2023;12(3):594. 36978841 10.3390/antiox12030594 PMC10045774

[36] Morry J, Ngamcherdtrakul W, Yantasee W. Oxidative stress in cancer and fibrosis: Opportunity for therapeutic intervention with antioxidant compounds, enzymes, and nanoparticles. Redox Biol. 2017;11:240–253. 28012439 10.1016/j.redox.2016.12.011 PMC5198743

[37] Singh S. Antioxidant nanozymes as next-generation therapeutics to free radical-mediated inflammatory diseases: A comprehensive review. Int J Biol Macromol. 2024;260: Article 129374. 38242389 10.1016/j.ijbiomac.2024.129374

[38] Han J, Wang J, Shi H, Li Q, Zhang S, Wu H, Li W, Gan L, Brown-Borg HM, Feng W, et al. Ultra-small polydopamine nanomedicine-enabled antioxidation against senescence. Mater Today Bio. 2023;19: Article 100544. 10.1016/j.mtbio.2023.100544 PMC9898451 36747580

[39] Nagendran V, Goveas LC, Vinayagam R, Varadavenkatesan T, Selvaraj R. Nanozymes in environmental remediation: A bibliometric and comprehensive review of their oxidoreductase-mimicking capabilities. Microchem J. 2024;207: Article 111748.

[40] Chowdhury S, Sanyal D, Sen S, Uversky VN, Maulik U, Chattopadhyay K. Evolutionary analyses of sequence and structure space unravel the structural facets of SOD1. Biomolecules. 2019;9(12):826. 31817166 10.3390/biom9120826 PMC6995586

[41] Zhang Y, Zheng L, Yun L, Ji L, Li G, Ji M, Shi Y, Zheng X. Catalase (CAT) Gene family in wheat (Triticum aestivum L.): Evolution, expression pattern and function analysis. Int J Mol Sci 2022;23(1):542. 35008967 10.3390/ijms23010542 PMC8745605

[42] Adriani PP, de Paiva FCR, de Oliveira GS, Leite AC, Sanches AS, Lopes AR, Dias MVB, Chambergo FS. Structural and functional characterization of the glutathione peroxidase-like thioredoxin peroxidase from the fungus Trichoderma reesei. Int J Biol Macromol. 2021;167:93–100. 33259843 10.1016/j.ijbiomac.2020.11.179

[43] Zámocký M, Koller F. Understanding the structure and function of catalases: Clues from molecular evolution and in vitro mutagenesis. Prog Biophys Mol Biol. 1999;72(1):19–66. 10446501 10.1016/s0079-6107(98)00058-3

[44] Bielski BHJ, Cabelli DE, Arudi RL, Ross AB. Reactivity of HO_2_/O^−^_2_ radicals in aqueous solution. J Phys Chem Ref Data. 1985;14(4):1041–1100.

[45] Wu J, Wang X, Wang Q, Lou Z, Li S, Zhu Y, Qin L, Wei H. Nanomaterials with enzyme-like characteristics (nanozymes): Next-generation artificial enzymes (II). Chem Soc Rev. 2019;48(4):1004–1076. 30534770 10.1039/c8cs00457a

[46] Zhang J, Wang Z, Lin X, Gao X, Wang Q, Huang R, Ruan Y, Xu H, Tian L, Ling C, et al. Mn−Ce symbiosis: Nanozymes with multiple active sites facilitate scavenging of reactive oxygen species (ROS) based on electron transfer and confinement anchoring. Angew Chem Int Ed Engl. 2025;64(4): Article e202416686. 39327805 10.1002/anie.202416686

[47] Yu Y, Zhao X, Zheng Y, Xia D, Liu Y. Core-shell structured CeO_2_@ZIF-8 nanohybrids regulating the Ce(III)/Ce(IV) valence conversion to enhance ROS-scavenging capacity for periodontitis treatment. Biomaterials. 2026;325: Article 123588. 40759062 10.1016/j.biomaterials.2025.123588

[48] Zhang Y, Zhang Y, Liang R, Zou J, Pei R, Chen X. Targeted ROS scavenging for disease therapies using nanomaterials. Adv Mater. 2025;37(50): Article e04435. 40844075 10.1002/adma.202504435

[49] Lin W, Gu S, Zhang X, Li K, Zhao D, Ma B, Pan C, Xu Z, Liu T, Wang H, et al. Remodeling adipocytes’ lipid metabolism with a polycation loaded enzyme-active framework reverses osteoporotic bone marrow. Nat Commun. 2025;16(1):8009. 40866384 10.1038/s41467-025-63376-4 PMC12391307

[50] Gao W, He J, Chen L, Meng X, Ma Y, Cheng L, Tu K, Gao X, Liu C, Zhang M, et al. Deciphering the catalytic mechanism of superoxide dismutase activity of carbon dot nanozyme. Nat Commun. 2023;14(1):160. 36631476 10.1038/s41467-023-35828-2 PMC9834297

[51] Zhang R, Chen L, Liang Q, Xi J, Zhao H, Jin Y, Gao X, Yan X, Gao L, Fan K. Unveiling the active sites on ferrihydrite with apparent catalase-like activity for potentiating radiotherapy. Nano Today. 2021;41: Article 101317.

[52] Yuan B, Tan Z, Guo Q, Shen X, Zhao C, Chen JL, Peng Y-K. Regulating the H_2_O_2_ activation pathway on a well-defined CeO_2_ nanozyme allows the entire steering of its specificity between associated enzymatic reactions. ACS Nano. 2023;17(17):17383–17393. 37578491 10.1021/acsnano.3c05409

[53] Yuan B, Chou H-L, Peng Y-K. Disclosing the origin of transition metal oxides as peroxidase (and catalase) mimetics. ACS Appl Mater Interfaces. 2022;14(20):22728–22736. 34634906 10.1021/acsami.1c13429

[54] Lai Y, Wang J, Yue N, Zhang Q, Wu J, Qi W, Su R. Glutathione peroxidase-like nanozymes: Mechanism, classification, and bioapplication. Biomater Sci. 2023;11(7):2292–2316. 36790050 10.1039/d2bm01915a

[55] Kelm JE, Bredar ARC, Rountree KJ, Dempsey JL. The relationship between surface defectivity of CdSe quantum dots and interfacial charge transfer studied by cyclic voltammetry. J Phys Chem C. 2025;129(23):10601–10612.

[56] Georgiou-Siafis SK, Tsiftsoglou AS. The key role of GSH in keeping the redox balance in mammalian cells: Mechanisms and significance of GSH in detoxification via formation of conjugates. Antioxidants. 2023;12(11):1953. 38001806 10.3390/antiox12111953 PMC10669396

[57] Chen L, Ding C, Chai K, Yang B, Chen W, Zeng J, Xu W, Huang Y. Nanohole-array induced metallic molybdenum selenide nanozyme for photoenhanced tumor-specific therapy. ACS Nano. 2023;17(18):18148–18163. 37713431 10.1021/acsnano.3c05000

[58] Ishii T, Warabi E, Yanagawa T. Novel roles of peroxiredoxins in inflammation, cancer and innate immunity. J Clin Biochem Nutr. 2012;50(2):91–105. 22448089 10.3164/jcbn.11-109 PMC3303482

[59] Seco-Cervera M, González-Cabo P, Pallardó FV, Romá-Mateo C, García-Giménez JL. Thioredoxin and glutaredoxin systems as potential targets for the development of new treatments in Friedreich’s ataxia. Antioxidants. 2020;9(12): Article 1257. 33321938 10.3390/antiox9121257 PMC7763308

[60] Sastre S, Manta B, Semelak JA, Estrin D, Trujillo M, Radi R, Zeida A. Catalytic mechanism of Mycobacterium tuberculosis methionine sulfoxide reductase A. Biochemistry. 2024;63(4):533–544. 38286790 10.1021/acs.biochem.3c00504

[61] Jomova K, Alomar SY, Alwasel SH, Nepovimova E, Kuca K, Valko M. Several lines of antioxidant defense against oxidative stress: Antioxidant enzymes, nanomaterials with multiple enzyme-mimicking activities, and low-molecular-weight antioxidants. Arch Toxicol. 2024;98(5):1323–1367. 38483584 10.1007/s00204-024-03696-4 PMC11303474

[62] Han D, Li X, Zhang L. Recent advancements for defect engineering in nanozymes. Coord Chem Rev. 2026;546: Article 217067.

[63] Li Y, Zeng Z, Tong J, Yang T, Liu G, Feng B, Zhang P, Liu X, Qing T. Surface ligand-regulated nanointerfaces: Enhancing the catalytic activity and selectivity of platinum nanozymes for biomedical applications. Appl Surf Sci. 2024;655: Article 159695.

[64] Deng Z, Zhang Y, Li R, Zhu Y, Xu C, Gao B, Wang W, Ding C, He B, Zhu X, et al. Honeysuckle-derived carbon dots with robust catalytic and pharmacological activities for mitigating lung inflammation by inhibition of caspase11/GSDMD-dependent pyroptosis. Adv Funct Mater. 2025;35:2418683.

[65] Gao X, Yang X, Deng C, Chen Y, Bian Y, Zhang X, Jin Y, Zhang J, Liang XJ. A mitochondria-targeted nanozyme with enhanced antioxidant activity to prevent acute liver injury by remodeling mitochondria respiratory chain. Biomaterials. 2025;318: Article 123133. 39879841 10.1016/j.biomaterials.2025.123133

[66] Huang X, Zhang K, Peng B, Wang G, Muhler M, Wang F. Ceria-based materials for thermocatalytic and photocatalytic organic synthesis. ACS Catal. 2021;11(15):9618–9678.

[67] Liu C, Fan W, Cheng W-X, Gu Y, Chen Y, Zhou W, Yu X-F, Chen M, Zhu M, Fan K, et al. Red emissive carbon dot superoxide dismutase nanozyme for bioimaging and ameliorating acute lung injury. Adv Funct Mater. 2023;33(19):2213856.

[68] Wang Q, Cheng C, Zhao S, Liu Q, Zhang Y, Liu W, Zhao X, Zhang H, Pu J, Zhang S, et al. A valence-engineered self-cascading antioxidant nanozyme for the therapy of inflammatory bowel disease. Angew Chem Int Ed Engl. 2022;61(27): Article e202201101. 35452169 10.1002/anie.202201101

[69] Zhao H, Zhao H, Li M, Tang Y, Xiao X, Cai Y, He F, Huang H, Zhang Y, Li J. Twin defect-rich Pt ultrathin nanowire nanozymes alleviate inflammatory skin diseases by scavenging reactive oxygen species. Redox Biol. 2024;70: Article 103055. 38290385 10.1016/j.redox.2024.103055 PMC10844124

[70] Qiu Y, Wu Y, Wei X, Luo X, Jiang W, Zheng L, Gu W, Zhu C, Yamauchi Y. Improvement in ORR durability of Fe single-atom carbon catalysts hybridized with CeO_2_ nanozyme. Nano Lett. 2024;24(29):9034–9041. 38990087 10.1021/acs.nanolett.4c02178

[71] Xie Y, Li Q, Huang L, Wang P, Wang T, Bai M, Guo J, Geng W, Wang X, Qiao W, et al. Spatial configuration-guided design of covalent organic framework-based artificial metalloantioxidases for inhibiting inflammatory cascades and regulating bone homeostasis. J Am Chem Soc. 2025;147(22):19113–19131. 40405440 10.1021/jacs.5c04242

[72] Zhang Y, Gao W, Ma Y, Cheng L, Zhang L, Liu Q, Chen J, Zhao Y, Tu K, Zhang M, et al. Integrating Pt nanoparticles with carbon nanodots to achieve robust cascade superoxide dismutase-catalase nanozyme for antioxidant therapy. Nano Today. 2023;49: Article 101768.

[73] Li L, Liu X, Liu G, Xu S, Hu G, Wang L. Valence-engineered catalysis-selectivity regulation of molybdenum oxide nanozyme for acute kidney injury therapy and post-cure assessment. Nat Commun. 2024;15(1):8720. 39379388 10.1038/s41467-024-53047-1 PMC11461881

[74] Li G, Guo X, Luo J, Guo J, Xiao S, Yang X, Shi X, Xiang J, Yang J, Ma T, et al. Optimizing the d-band center of Pd/CoPcS-Ti3C2Tx to enhance SOD/CAT-mimicking activities in the treatment of osteoarthritis. Nano Today. 2024;56: Article 102250.

[75] Chu D, Hu T, Cui H, Yang L, Li Z, Tan C, Li J. Amorphous layered double hydroxide-based nano-enzyme eye drops against dry eye disease by inhibiting mitochondrial damage and pyroptosis. J Nanobiotechnol. 2025;23(1):688. 10.1186/s12951-025-03727-x PMC12548265 41126264

[76] Qiao Q, Liu Z, Hu F, Xu Z, Kuang Y, Li C. A novel Ce─Mn heterojunction-based multi-enzymatic nanozyme with cancer-specific enzymatic activity and photothermal capacity for efficient tumor combination therapy. Adv Funct Mater. 2025;35(6): Article 2414837.

[77] Le W, Sun Z, Li T, Cao H, Yang C, Mei T, Zhang L, Wang Y, Jia W, Sun W, et al. Antioxidant nanozyme-engineered mesenchymal stem cells for in vivo MRI tracking and synergistic therapy of myocardial infarction. Adv Funct Mater. 2024;34(23): Article 2314328.

[78] Yu X, Wang L, Zhu Z, Han X, Zhang J, Wang A, Ding L, Liu J. Piezoelectric effect modulates nanozyme activity: Underlying mechanism and practical application. Small. 2023;19(52): Article 2304818. 10.1002/smll.202304818 37635126

[79] Yu B, Sun W, Lin J, Fan C, Wang C, Zhang Z, Wang Y, Tang Y, Lin Y, Zhou D. Using Cu-based metal–organic framework as a comprehensive and powerful antioxidant nanozyme for efficient osteoarthritis treatment. Adv Sci. 2024;11: Article 2307798. 10.1002/advs.202307798 PMC10987124 38279574

[80] Guo L, Zhang H, Zhao Z, Zhang S, Hu W. Synthesis of Co/Ce dual active sites single-atom cerium nanozyme with the synergistic effect and peroxidase-like activity for total antioxidant capacity evaluation. Anal Chem. 2025;97(30):16203–16210. 40695526 10.1021/acs.analchem.5c01219

[81] Li Z, Fan X, Liu Y, Yue M, Wu T, Wang X, Jiang W, Fan K. Engineering mild-photothermal responsive and NO donor Prussian blue nanozymes using mild synthesis for inflammation regulation and bacterial eradication in periodontal disease. Adv Mater. 2025;37(6): Article 2409840. 10.1002/adma.202409840 39690880

[82] Li Y, Li Q, Zhu Y, Huang C, Li H, Deng Z, Xu C, Wang W, Chen L, Zhang S, et al. Medicinal plant-derived carbon dots nanozymes ameliorate ulcerative colitis via anti-inflammatory, antioxidant, and gut barrier-protective effects. ACS Appl Mater Interfaces. 2025;17(30):42751–42766. 40611517 10.1021/acsami.5c08068

[83] Liu J, Huang X, Zhang F, Luo X, Yu W, Li C, Qiu Z, Liu Y, Xu Z. Metal-free multifunctional nanozymes mimicking endogenous antioxidant system for acute kidney injury alleviation. Chem Eng J. 2023;477: Article 147048.

[84] Zeng J, Ding C, Chen L, Yang B, Li M, Wang X, Su F, Liu C, Huang Y. Multienzyme-mimicking Au@Cu_2_O with complete antioxidant capacity for reactive oxygen species scavenging. ACS Appl Mater Interfaces. 2023;15(1):378–390. 36594213 10.1021/acsami.2c16995

[85] Zhao S, Ling J, Wang N, Ouyang X-K. Cerium dioxide nanozyme doped hybrid hydrogel with antioxidant and antibacterial abilities for promoting diabetic wound healing. Chem Eng J. 2024;497: Article 154517.

[86] Jiang D, Yang B, Shi J. Nanocatalytic antioxidation enables aortic endothelial repair and aortic aneurysm treatment. Nano Lett. 2025;25(20):8176–8185. 40357560 10.1021/acs.nanolett.5c01054

[87] Xiao S, Huang S, Wang M, Wang T, Han M, Deng Y, Geng W, Cheng L, Wang X, Ma L, et al. Biocatalytic and redox-regulated nanoarchitectures for precision inflammation and immune homeostasis modulation to combat rheumatoid arthritis. Adv Mater. 2025;37(33):2502147. 10.1002/adma.202502147 40451749

[88] Deng Z, Li C, Zhang Y, Zhu Y, Xu C, Gao B, Zhang M, Stenzel MH, Fan K, Zhang M, et al. Deciphering the catalytic and pharmacological mechanisms of Coptis chinensis herbzymes to renovate intestinal microenvironment for colitis alleviation. Bmemat. 2025; Article e70031.

[89] Krishnendu MR, Singh S. Reactive oxygen species: Advanced detection methods and coordination with nanozymes. Chem Eng J. 2025;511: Article 161296.

[90] Chen Q, Duan X, Yu Y, Ni R, Song G, Yang X, Zhu L, Zhong Y, Zhang K, Qu K, et al. Target functionalized carbon dot nanozymes with dual-model photoacoustic and fluorescence imaging for visual therapy in atherosclerosis. Adv Sci. 2024;11(6):2307441. 10.1002/advs.202307441 PMC10853701 38145362

[91] McHugh EA, Liopo AV, Mendoza K, Robertson CS, Wu G, Wang Z, Chen W, Beckham JL, Derry PJ, Kent TA, et al. Oxidized activated charcoal nanozymes: Synthesis, and optimization for in vitro and in vivo bioactivity for traumatic brain injury. Adv Mater. 2024;36(10):2211239. 10.1002/adma.202211239 PMC10509328 36940058

[92] Wu G, Berka V, Derry PJ, Mendoza K, Kakadiaris E, Roy T, Kent TA, Tour JM, Tsai A-L. Critical comparison of the superoxide dismutase-like activity of carbon antioxidant nanozymes by direct superoxide consumption kinetic measurements. ACS Nano. 2019;13(10):11203–11213. 31509380 10.1021/acsnano.9b04229 PMC6832779

[93] Yang Y, Zeng Q, Zhao C, Shi J, Wang W, Liang Y, Li C, Guan Q, Chen B, Li W. Metal-free antioxidant nanozyme incorporating bioactive hydrogel as an antioxidant scaffold for accelerating bone reconstruction. Biomaterials. 2025;320: Article 123285. 40127506 10.1016/j.biomaterials.2025.123285

[94] Huang X, Wang J, Wang H, Ma R, Ling Z, Chen K, Xu Z, Ren J, Wu X, Zhang Q, et al. Silver-catechol dynamic redox chemistry provides hydrogel dressings with sustained antioxidant and antibacterial activity for chronic wound care. ACS Nano. 2025;19(24):22270–22290. 40504008 10.1021/acsnano.5c04690

[95] Xia F, Hu X, Zhang B, Wang X, Guan Y, Lin P, Ma Z, Sheng J, Ling D, Li F. Ultrasmall ruthenium nanoparticles with boosted antioxidant activity upregulate regulatory T cells for highly efficient liver injury therapy. Small. 2022;18(29):2201558. 10.1002/smll.202201558 35748217

[96] Xie Y, Xiao S, Huang L, Guo J, Bai M, Gao Y, Zhou H, Qiu L, Cheng C, Han X. Cascade and ultrafast artificial antioxidases alleviate inflammation and bone resorption in periodontitis. ACS Nano. 2023;17(15):15097–15112. 37378617 10.1021/acsnano.3c04328

[97] Chen Z, Chen P, Zhu Y, Qian J, Huang X, Zhang W, Zhang H, Mo Q, Lu Y, Zhang Y. 2D cobalt oxyhydroxide nanozymes inhibit inflammation by targeting the NLRP3 inflammasome. Adv Funct Mater. 2023;33(27): Article 2214693.

[98] Sun Z, Muhammad F, Qiao C, Gong W, Wang Z, Liu Y, Yu X, Dong J, Lv J, Cheng X, et al. Templated synthesis of hollow RuO_2_ nanospheres for alleviating metal wear particle-induced osteoclast activation and bone loss. Small. 2025;21: Article 2406210. 10.1002/smll.202406210 39623799

[99] Wei YJ, Chen H, Zhou ZW, Liu CX, Cai CX, Li J, Yu XQ, Zhang J, Liu YH, Wang N. Kill two birds with one stone: Dual-metal MOF-nanozyme-decorated hydrogels with ROS-scavenging, oxygen-generating, and antibacterial abilities for accelerating infected diabetic wound healing. Small. 2024;20(48): Article e2403679. 39240068 10.1002/smll.202403679

[100] Xiang K, Wu H, Liu Y, Wang S, Li X, Yang B, Zhang Y, Ma L, Lu G, He L, et al. MOF-derived bimetallic nanozyme to catalyze ROS scavenging for protection of myocardial injury. Theranostics. 2023;13(8):2721–2733. 37215581 10.7150/thno.83543 PMC10196836

[101] Yu Y, Zhang Y, Wang Y, Chen W, Guo Z, Song N, Liang M. Multiscale structural design of MnO_2_@GO superoxide dismutase nanozyme for protection against antioxidant damage. Nano Res. 2023;16:10763–10769.

[102] Yang L, Dong S, Gai S, Yang D, Ding H, Feng L, Yang G, Rehman Z, Yang P. Deep insight of design, mechanism, and cancer theranostic strategy of nanozymes. Nano-Micro Lett. 2023;16(1):28. 10.1007/s40820-023-01224-0 PMC10663430 37989794

[103] Xie M, Du J, Jia M, Nie X, Zhang X, Hu Y, Nian B. Computer-aided techniques in the engineering of enzyme binding pockets: New perspectives and frontiers. J Agric Food Chem. 2025;73(33):20600–20615. 40790341 10.1021/acs.jafc.5c05979

[104] Huang F, Lu X, Kuai L, Ru Y, Jiang J, Song J, Chen S, Mao L, Li Y, Li B, et al. Dual-site biomimetic cu/Zn-MOF for atopic dermatitis catalytic therapy via suppressing FcγR-mediated phagocytosis. J Am Chem Soc. 2024;146(5):3186–3199. 38266487 10.1021/jacs.3c11059

[105] Yang B, Hu C, Zhang Y, Jiang D, Lin P, Qiu S, Shi J, Wang L. Biomimetic-structured cobalt nanocatalyst suppresses aortic dissection progression by catalytic antioxidation. J Am Chem Soc. 2024;146(25):17201–17210. 38874405 10.1021/jacs.4c03344

[106] Lu X, Kuai L, Huang F, Jiang J, Song J, Liu Y, Chen S, Mao L, Peng W, Luo Y, et al. Single-atom catalysts-based catalytic ROS clearance for efficient psoriasis treatment and relapse prevention via restoring ESR1. Nat Commun. 2023;14(1):6767. 37880231 10.1038/s41467-023-42477-y PMC10600197

[107] Wang J, Wang Y, Xiaohalati X, Su Q, Liu J, Cai B, Yang W, Wang Z, Wang L. A bioinspired manganese-organic framework ameliorates ischemic stroke through its intrinsic nanozyme activity and upregulating endogenous antioxidant enzymes. Adv Sci. 2023;10(20):2206854. 10.1002/advs.202206854 PMC10369237 37129343

[108] Zhao Y, Zhao S, Du Y, Gao Z, Li Y, Ma H, Li H, Ren X, Fan Q, Wu D, et al. Inverse oxide/alloy-structured nanozymes with NIR-triggered enzymatic cascade regulation of ROS homeostasis for efficient wound healing. Adv Mater. 2025;37(14):2418731. 10.1002/adma.202418731 39995376

[109] Jiang D, Yang B, Shi J. Antioxidative therapy of alcoholic liver injury by amorphous two-dimensional cobalt hydroxide nanocatalyst. Angew Chem Int Ed Engl. 2025;64(1): Article e202412031. 39513490 10.1002/anie.202412031

[110] Jiang C, Sun M, Wang Y, Dong C, Yu Y, Wang G, Lu Y, Chen Z. Coordination engineering in Fe-Mn dual-atom nanozyme: Yielding ROS storm to efficiently promote wound healing. Adv Funct Mater. 2025;35(29):2424599.

[111] Guo W, Ji M, Li Y, Qian M, Qin Y, Li W, Nie H, Lv W, Jiang G, Huang R, et al. Iron ions-sequestrable and antioxidative carbon dot-based nano-formulation with nitric oxide release for Parkinson’s disease treatment. Biomaterials. 2024;309: Article 122622. 38797119 10.1016/j.biomaterials.2024.122622

[112] Zhang R, Xue B, Tao Y, Zhao H, Zhang Z, Wang X, Zhou X, Jiang B, Yang Z, Yan X, et al. Edge-site engineering of defective Fe–N4 nanozymes with boosted catalase-like performance for retinal vasculopathies. Adv Mater. 2022;34(39):2205324. 10.1002/adma.202205324 35953446

[113] Liu Q, Xie W, Liao J, Li G, Wang H, Yang H. Montmorillonite modulates manganese d-band center to enhance cascade reaction activity against inflammatory bowel disease. Adv Funct Mater. 2025;35(46):2502389.

[114] Wang Z, Wu J, Zheng J-J, Shen X, Yan L, Wei H, Gao X, Zhao Y. Accelerated discovery of superoxide-dismutase nanozymes via high-throughput computational screening. Nat Commun. 2021;12(1):6866. 34824234 10.1038/s41467-021-27194-8 PMC8616946

[115] del Bosque A, Fernández-Arias P, Vergara D. Machine learning for nanomaterial discovery and design. Mach Learn Knowl Extract. 2026;8(1):10.

[116] Wei Y, Wu J, Wu Y, Liu H, Meng F, Liu Q, Midgley AC, Zhang X, Qi T, Kang H, et al. Prediction and design of nanozymes using explainable machine learning. Adv Mater. 2022;34(27):2201736. 10.1002/adma.202201736 35487518

[117] Sun L, Hu J, Yang Y, Wang Y, Wang Z, Gao Y, Nie Y, Liu C, Kan H. ChatGPT combining machine learning for the prediction of nanozyme catalytic types and activities. J Chem Inf Model. 2024;64(17):6736–6744. 38829968 10.1021/acs.jcim.4c00600

[118] Zhao X, Yu Y, Xu X, Zhang Z, Chen Z, Gao Y, Zhong L, Chen J, Huang J, Qin J, et al. Machine learning-assisted high-throughput screening of nanozymes for ulcerative colitis. Adv Mater. 2025;37(9):2417536. 10.1002/adma.202417536 39801185

[119] Chen S, Huang F, Mao L, Zhang Z, Lin H, Yan Q, Lu X, Shi J. High Fe-loading single-atom catalyst boosts ROS production by density effect for efficient antibacterial therapy. Nano-Micro Lett. 2024;17(1):32. 10.1007/s40820-024-01522-1 PMC11450126 39363132

[120] Zhao R, Li L, Yan Y, Xue X, Huo Y, Liu T, Wang H, Liu C, Zhang S, Sun S, et al. Single-atom modulated biocatalytic selectivity on WSe2 clusters. ACS Appl Mater Interfaces. 2025;17(26):38367–38378. 40544354 10.1021/acsami.5c07454

[121] Li L, Li H, Shi L, Shi L, Li T. Tin porphyrin-based nanozymes with unprecedented superoxide dismutase-mimicking activities. Langmuir. 2022;38(23):7272–7279. 35638128 10.1021/acs.langmuir.2c00778

[122] Zhang S, Li Y, Sun S, Liu L, Mu X, Liu S, Jiao M, Chen X, Chen K, Ma H, et al. Single-atom nanozymes catalytically surpassing naturally occurring enzymes as sustained stitching for brain trauma. Nat Commun. 2022;13(1):4744. 35961961 10.1038/s41467-022-32411-z PMC9374753

[123] Liu X, Xu H, Peng H, Wan L, Di D, Qin Z, He L, Lu J, Wang S, Zhao Q. Advances in antioxidant nanozymes for biomedical applications. Coord Chem Rev. 2024;502: Article 215610.

[124] Zhang S, Gao XJ, Ma Y, Song K, Ge M, Ma S, Zhang L, Yuan Y, Jiang W, Wu Z, et al. A bioinspired sulfur–Fe–heme nanozyme with selective peroxidase-like activity for enhanced tumor chemotherapy. Nat Commun. 2024;15:10605. 39638998 10.1038/s41467-024-54868-w PMC11621791

[125] He Y, Peng E, Ba X, Wu J, Deng W, Huang Q, Tong Y, Shang H, Zhong Z, Liu X, et al. ROS responsive cerium oxide biomimetic nanoparticles alleviates calcium oxalate crystals induced kidney injury via suppressing oxidative stress and M1 macrophage polarization. Small. 2025;21(3):2405417. 10.1002/smll.202405417 39629501

[126] He L, Li Z, Gu M, Li Y, Yi C, Jiang M, Yu X, Xu L. Intelligent carbon dots with switchable photo-activated oxidase-mimicking activity and pH responsive antioxidant activity adaptive to the wound microenvironment for selective antibacterial therapy. Adv Sci. 2024;11(40):2406681. 10.1002/advs.202406681 PMC11516101 39225540

[127] Jiang W, Hou X, Qi Y, Wang Z, Liu Y, Gao XJ, Wu T, Guo J, Fan K, Shang W. pH-activatable pre-nanozyme mediated H2S delivery for endo-exogenous regulation of oxidative stress in acute kidney injury. Adv Sci. 2024;11(18):2303901. 10.1002/advs.202303901 PMC11095207 38445847

[128] Xu Y, Luo Y, Weng Z, Xu H, Zhang W, Li Q, Liu H, Liu L, Wang Y, Liu X, et al. Microenvironment-responsive metal-phenolic nanozyme release platform with antibacterial, ROS scavenging, and osteogenesis for periodontitis. ACS Nano. 2023;17(19):18732–18746. 37768714 10.1021/acsnano.3c01940

[129] Zhang Y, Zhang X, Wu X, Zhao Y. Photo-responsive polydopamine nanoenzyme microneedles with oxidative stress regulation ability for atopic dermatitis treatment. Nano Today. 2024;56: Article 102241.

[130] Ding Q, Liu H, Yan L, Chen L, Chen Y, Kim JS, Mei L. Engineering a multifunctional nanozyme platform for synergistic melanoma therapy: Integrating enzyme activity, immune activation, and low-temperature photothermal effects. Angew Chem Int Ed Engl. 2025;64(32): Article e202505911. 40454607 10.1002/anie.202505911 PMC12322653

[131] Lv W, Jiang G, Lin X, Qian M, Huang R, Li Z, Liu H, Lin D, Wang Y. An unusual application of multifunctional nanozyme derived from COF: Augmenting chemoimmunotherapy while attenuating cardiotoxicity. Adv Funct Mater. 2025;35(2):2412862.

[132] Wu H, Xia F, Zhang L, Fang C, Lee J, Gong L, Gao J, Ling D, Li F. A ROS-sensitive nanozyme-augmented photoacoustic nanoprobe for early diagnosis and therapy of acute liver failure. Adv Mater. 2022;34(7):2108348. 10.1002/adma.202108348 34839560

[133] Wei J, Zhou Z, Pu X, Wu X, Zhang Y, Zhong T, Huang W, Zhong Z, Wang X. Cold beer-inspired multifunctional nanozyme for ischemic stroke with rapid thrombus clearance and long-lasting hydrogen therapy. Nano Today. 2025;61: Article 102636.

[134] Zhou H, He J, Liu R, Cheng J, Yuan Y, Mao W, Zhou J, He H, Liu Q, Tan W, et al. Microenvironment-responsive metal-phenolic network release platform with ROS scavenging, anti-pyroptosis, and ECM regeneration for intervertebral disc degeneration. Bioact Mater. 2024;37:51–71. 38515609 10.1016/j.bioactmat.2024.02.036 PMC10954684

[135] Singh N, NaveenKumar SK, Geethika M, Mugesh G. A cerium vanadate nanozyme with specific superoxide dismutase activity regulates mitochondrial function and ATP synthesis in neuronal cells. Angew Chem Int Ed Engl. 2021;60(6):3121–3130. 33079465 10.1002/anie.202011711

[136] Liu Y, Cheng Y, Zhang H, Zhou M, Yu Y, Lin S, Jiang B, Zhao X, Miao L, Wei C-W, et al. Integrated cascade nanozyme catalyzes in vivo ROS scavenging for anti-inflammatory therapy. Sci Adv. 6: Article eabb2695. 10.1126/sciadv.abb2695 PMC7439611 32832640

[137] Shen H, Fu Y, Liu F, Zhang W, Yuan Y, Yang G, Yang M, Li L. AuCePt porous hollow cascade nanozymes targeted delivery of disulfiram for alleviating hepatic insulin resistance. J Nanobiotechnol. 2024;22(1):660. 10.1186/s12951-024-02880-z PMC11515139 39456019

[138] Wang H, Yang B, Du C, Zheng H, Zhang X, Chen J. In situ cascade catalytic polymerization of dopamine based on Pt NPs/CoSAs@NC nanoenzyme for constructing highly sensitive photocurrent-polarity-switching PEC biosensing platform. Small. 2025;21(7):2409990. 10.1002/smll.202409990 39760264

[139] Liu J, Wei J, Xiao S, Yuan L, Liu H, Zuo Y, Li Y, Li J. Multienzyme-activity sulfur quantum dot nanozyme-mediated cascade reactions in whole-stage symptomatic therapy of infected bone defects. ACS Nano. 2025;19(7):6858–6875. 39936642 10.1021/acsnano.4c12343

[140] Liu W, Zhang Y, Wei G, Zhang M, Li T, Liu Q, Zhou Z, Du Y, Wei H. Integrated cascade nanozymes with antisenescence activities for atherosclerosis therapy. Angew Chem Int Ed Engl. 2023;62(33): Article e202304465. 37338457 10.1002/anie.202304465

[141] Luo T, Yang H, Wang R, Pu Y, Cai Z, Zhao Y, Bi Q, Lu J, Jin R, Nie Y, et al. Bifunctional cascading nanozymes based on carbon dots promotes photodynamic therapy by regulating hypoxia and glycolysis. ACS Nano. 2023;17(17):16715–16730. 37594768 10.1021/acsnano.3c03169

[142] Thao NTM, Do HDK, Nam NN, Tran NKS, Dan TT, Trinh KTL. Antioxidant Nanozymes: Mechanisms, activity manipulation, and applications. Micromachines. 2023;14(5):1017. 37241640 10.3390/mi14051017 PMC10220853

[143] Feng K, Wang G, Wang S, Ma J, Wu H, Ma M, Zhang Y. Breaking the pH limitation of nanozymes: Mechanisms, methods, and applications. Adv Mater. 2024;36(31):2401619. 10.1002/adma.202401619 38615261

[144] Chen X, Liao J, Lin Y, Zhang J, Zheng C. Nanozyme’s catalytic activity at neutral pH: Reaction substrates and application in sensing. Anal Bioanal Chem. 2023;415(18):3817–3830. 36633622 10.1007/s00216-023-04525-w

[145] Xu D, Wu L, Yao H, Zhao L. Catalase-like nanozymes: Classification, catalytic mechanisms, and their applications. Small. 2022;18(37):2203400. 10.1002/smll.202203400 35971168

[146] Yang W, Yang X, Zhu L, Chu H, Li X, Xu W. Nanozymes: Activity origin, catalytic mechanism, and biological application. Coord Chem Rev. 2021;448: Article 214170.

[147] Jiang D, Ni D, Rosenkrans ZT, Huang P, Yan X, Cai W. Nanozyme: New horizons for responsive biomedical applications. Chem Soc Rev. 2019;48(14):3683–3704. 31119258 10.1039/c8cs00718g PMC6696937

[148] Zhu H, He H, Chen Z, Zhu J, Sun J, Zhang J. Adaptive ROS regulating nanomedicine for tumor catalytic and immunotherapy beyond oxidative overload. ACS Nano Med. 2026;1(3):564–593.

[149] Huang Q, Zhang H, Chen S, Wang Y, Zhou J. Ferroptosis in central nervous system injuries: Molecular mechanisms, diagnostic approaches, and therapeutic strategies. Front Cell Neurosci. 2025;19:1593963. 40766185 10.3389/fncel.2025.1593963 PMC12321900

[150] Goldsteins G, Hakosalo V, Jaronen M, Keuters MH, Lehtonen Š, Koistinaho J. CNS redox homeostasis and dysfunction in neurodegenerative diseases. Antioxidants. 2022;11(2):405. 35204286 10.3390/antiox11020405 PMC8869494

[151] Eroglu E, Harmanci N. Emerging molecular targets in neurodegenerative disorders: New avenues for therapeutic intervention. Basic Clin Pharmacol Toxicol. 2025;137(4): Article e70107. 40922457 10.1111/bcpt.70107

[152] Jiang Y, Rong H, Wang Y, Liu S, Xu P, Luo Z, Guo L, Zhu T, Rong H, Wang D, et al. Single-atom cobalt nanozymes promote spinal cord injury recovery by anti-oxidation and neuroprotection. Nano Res. 2023;16:9752–9759.

[153] Chen L, Hu Y, Cheng Y, Wang H. A hydroxyquinoline polymer with excellent amyloidosis inhibition and protein delivery ability to combat amyloid-β-mediated neurotoxicity. Nano Lett. 2024;24(41):12882–12890. 10.1021/acs.nanolett.4c03275 39352880

[154] Dias V, Junn E, Mouradian MM. The role of oxidative stress in Parkinson’s disease. J Parkinsons Dis. 2013;3(4):461–491. 24252804 10.3233/JPD-130230 PMC4135313

[155] Jiang W, Li Q, Zhang R, Li J, Lin Q, Li J, Zhou X, Yan X, Fan K. Chiral metal-organic frameworks incorporating nanozymes as neuroinflammation inhibitors for managing Parkinson’s disease. Nat Commun. 2023;14(1):8137. 38065945 10.1038/s41467-023-43870-3 PMC10709450

[156] Liu Y, Liu Y, Shi P, Hu X, Fan X, Wu Y, Pan J, Bai Q, Li Q. Single-atom nanozyme liposome-integrated microneedles for in situ drug delivery and anti-inflammatory therapy in Parkinson’s disease. J Nanobiotechnol. 2024;22(1):643. 10.1186/s12951-024-02924-4 PMC11490154 39427214

[157] Wu L, Xiong X, Wu X, Ye Y, Jian Z, Zhi Z, Gu L. Targeting oxidative stress and inflammation to prevent ischemia-reperfusion injury. Front Mol Neurosci. 2020;13:28. 32194375 10.3389/fnmol.2020.00028 PMC7066113

[158] Tian R, Ma H, Ye W, Li Y, Wang S, Zhang Z, Liu S, Zang M, Hou J, Xu J, et al. Se-containing MOF coated dual-Fe-atom nanozymes with multi-enzyme cascade activities protect against cerebral ischemic reperfusion injury. Adv Funct Mater. 2022;32(36):2204025.

[159] Lakhan SE, Kirchgessner A, Hofer M. Inflammatory mechanisms in ischemic stroke: Therapeutic approaches. J Transl Med. 2009;7:97. 19919699 10.1186/1479-5876-7-97 PMC2780998

[160] Wang Z, Zhao Y, Hou Y, Tang G, Zhang R, Yang Y, Yan X, Fan K. A thrombin-activated peptide-templated nanozyme for remedying ischemic stroke via thrombolytic and neuroprotective actions. Adv Mater. 2024;36(10):2210144. 10.1002/adma.202210144 36730098

[161] Dhapola R, Beura SK, Sharma P, Singh SK, HariKrishnaReddy D. Oxidative stress in Alzheimer’s disease: Current knowledge of signaling pathways and therapeutics. Mol Biol Rep. 2024;51(1):48. 38165499 10.1007/s11033-023-09021-z

[162] Yang J, Qin G, Liu Z, Zhang H, Du X, Ren J, Qu X. A Nanozyme-boosted MOF-CRISPR platform for treatment of Alzheimer’s disease. Nano Lett. 2024;24(32):9906–9915. 39087644 10.1021/acs.nanolett.4c02272

[163] Chávez MD, Tse HM. Targeting mitochondrial-derived reactive oxygen species in T cell-mediated autoimmune diseases. Front Immunol. 2021;12: Article 703972. 34276700 10.3389/fimmu.2021.703972 PMC8281042

[164] Kondo N, Kanai T, Okada M. Rheumatoid arthritis and reactive oxygen species: A review. Curr Issues Mol Biol. 2023;45(4):3000–3015. 37185721 10.3390/cimb45040197 PMC10137217

[165] He H, Zhang Q, Zhang Y, Qu S, Li B, Lin J, Lu X, Xie C. Injectable bioadhesive and lubricating hydrogel with polyphenol mediated single atom nanozyme for rheumatoid arthritis therapy. Nat Commun. 2025;16(1):2768. 40113809 10.1038/s41467-025-58059-z PMC11926226

[166] Li L, Peng P, Ding N, Jia W, Huang C, Tang Y. Oxidative stress, inflammation, gut dysbiosis: What can polyphenols do in inflammatory bowel disease? Antioxidants. 2023;12(4):967. 37107341 10.3390/antiox12040967 PMC10135842

[167] Li S, Chen Z, Wang M, Rao Y, Yang F, Liu M, Chu W, Yue W. L-arginine-modified selenium nanozymes targeting M1 macrophages for oral treatment of ulcerative colitis. Small. 2025;21:2408205. 10.1002/smll.202408205 39763139

[168] Liu K, Chen S, Geng X, Pei X, Xing H, Zhang X, Chang J, Yang W, Wu X. Multifunctional selenium-based metal polyphenol nanoparticles impede the pathological cross-talk between reactive oxygen species and inflammation in sepsis. ACS Mater Lett. 2024;6(6):2434–2445.

[169] Zhang Y, Liu W, Wei G, Liu Q, Shao G, Gu X, Cui X, Zhou Z, Wang Y, Zhao S, et al. Bioinspired nanozymes as nanodecoys for urinary tract infection treatment. ACS Nano. 2024;18(12):9019–9030. 38483200 10.1021/acsnano.3c12783

[170] Ito F, Sono Y, Ito T. Measurement and clinical significance of lipid peroxidation as a biomarker of oxidative stress: Oxidative stress in diabetes, atherosclerosis, and chronic inflammation. Antioxidants. 2019;8(3):72. 30934586 10.3390/antiox8030072 PMC6466575

[171] Gong G, Wan W, Zhang X, Chen X, Yin J. Management of ROS and regulatory cell death in myocardial ischemia–reperfusion injury. Mol Biotechnol. 2025;67(5):1765–1783. 38852121 10.1007/s12033-024-01173-y

[172] Ding C, Min J, Tan Y, Zheng L, Ma R, Zhao R, Zhao H, Ding Q, Chen H, Huo D. Combating atherosclerosis with chirality/phase dual-engineered nanozyme featuring microenvironment-programmed senolytic and senomorphic actions. Adv Mater. 2024;36(29):2401361. 10.1002/adma.202401361 38721975

[173] An H, Qiu X, Wang X, Du C, Guo X, Hou S, Xu M, Wang J, Cheng C, Ran H, et al. LIFU-unlocked endogenous H2S generation for enhancing atherosclerosis-specific gas-enzymatic therapy. Biomaterials. 2025;315: Article 122972. 39591768 10.1016/j.biomaterials.2024.122972

[174] He H, Han Q, Wang S, Long M, Zhang M, Li Y, Zhang Y, Gu N. Design of a multifunctional nanozyme for resolving the proinflammatory plaque microenvironment and attenuating atherosclerosis. ACS Nano. 2023;17(15):14555–14571. 37350440 10.1021/acsnano.3c01420

[175] Liu X, Chen B, Chen J, Wang X, Dai X, Li Y, Zhou H, Wu L-M, Liu Z, Yang Y. A cardiac-targeted nanozyme interrupts the inflammation-free radical cycle in myocardial infarction. Adv Mater. 2024;36(2):2308477. 10.1002/adma.202308477 37985164

[176] Glorieux C, Liu S, Trachootham D, Huang P. Targeting ROS in cancer: Rationale and strategies. Nat Rev Drug Discov. 2024;23(8):583–606. 38982305 10.1038/s41573-024-00979-4

[177] Yao Y, Xu R, Shao W, Tan J, Wang S, Chen S, Zhuang A, Liu X, Jia R. A novel nanozyme to enhance radiotherapy effects by lactic acid scavenging, ROS generation, and hypoxia mitigation. Adv Sci. 2024;11(26):2403107. 10.1002/advs.202403107 PMC11234405 38704679

[178] Fu X, Tang J, Wen P, Huang Z, Najafi M. Redox interactions-induced cardiac toxicity in cancer therapy. Arch Biochem Biophys. 2021;708: Article 108952. 34097901 10.1016/j.abb.2021.108952

[179] Huang X, Zhang F, Yang Y, Liu J, Tan X, Zhou P, Tang X, Hu J, Chen L, Yuan M, et al. Curcumin-copper complex nanoparticles as antioxidant nanozymes for acute kidney injury alleviation. Mater Today Bio. 2025;32: Article 101794. 10.1016/j.mtbio.2025.101794 PMC12088824 40391022

[180] Zhang Z, Cao Q, Xia Y, Cui C, Qi Y, Zhang Q, Wu Y, Liu J, Liu W. Combination of biodegradable hydrogel and antioxidant bioadhesive for treatment of breast cancer recurrence and radiation skin injury. Bioact Mater. 2024;31:408–421. 37692912 10.1016/j.bioactmat.2023.08.021 PMC10482898

[181] Sun W, Song J, Zhu C, Guo X, Jiang B-P, Gao C, Shen X-C. Multienzymatic hybrid metalloenzymes triggering cascade reactions-regulated tumor redox homeostasis and immunosuppressive microenvironment for catalytic immunotherapy. ACS Nano. 2025;19(26):24034–24051. 40570303 10.1021/acsnano.5c06592

[182] Singh TA, Sharma A, Tejwan N, Ghosh N, Das J, Sil PC. A state of the art review on the synthesis, antibacterial, antioxidant, antidiabetic and tissue regeneration activities of zinc oxide nanoparticles. Adv Colloid Interf Sci. 2021;295: Article 102495. 10.1016/j.cis.2021.102495 34375877

[183] Liu X, Yu L, Xiao A, Sun W, Wang H, Wang X, Zhou Y, Li C, Li J, Wang Y, et al. Analytical methods in studying cell force sensing: Principles, current technologies and perspectives. Regener Biomater. 2025;12:rbaf007. 10.1093/rb/rbaf007 PMC12057814 40337625

[184] Chao D, Dong Q, Yu Z, Qi D, Li M, Xu L, Liu L, Fang Y, Dong S. Specific nanodrug for diabetic chronic wounds based on antioxidase-mimicking MOF-818 nanozymes. J Am Chem Soc. 2022;144(51):23438–23447. 36512736 10.1021/jacs.2c09663

[185] Tai Q-D, Tang Y, Xie S-T, Ye Y-Y, Tang X, Lyu Q, Fan Z-J, Liao Y-H. Glucose-responsive nanozyme hydrogel for glycemic control and catalytic anti-infective therapy in diabetic wound healing. Mater Today Bio. 2025;35: Article 102405. 10.1016/j.mtbio.2025.102405 PMC12550175 41142415

[186] Yang D, Yuan M, Huang J, Xiang X, Pang H, Wei Q, Luo X, Cheng C, Qiu L, Ma L. Conjugated network supporting highly surface-exposed Ru site-based artificial antioxidase for efficiently modulating microenvironment and alleviating solar dermatitis. ACS Nano. 2024;18(4):3424–3437. 38227828 10.1021/acsnano.3c10552

[187] Yuan K-S, Deng C-C, Wang X-X, Li Y-C, Zhou C, Zhao C-R, Dai X-Z, Khan A-R, Zhang Z, Guidoin R, et al. Research advances and future perspectives of zinc-based biomaterials for additive manufacturing. Rare Metals. 2025;44:4376–4410.

[188] Deng C, Zhou Q, Zhang M, Li T, Chen H, Xu C, Feng Q, Wang X, Yin F, Cheng Y, et al. Bioceramic scaffolds with antioxidative functions for ROS scavenging and osteochondral regeneration. Adv Sci. 2022;9:2105727. 10.1002/advs.202105727 PMC9036007 35182053

[189] Jin Y, Zhang J, Chen X, Li F, Xue T, Yi K, Xu Y, Wang H, Lao Y-H, Chan HF, et al. 3D printing incorporating gold nanozymes with mesenchymal stem cell-derived hepatic spheroids for acute liver failure treatment. Biomaterials. 2025;315: Article 122895. 39461063 10.1016/j.biomaterials.2024.122895

[190] Lyu Z, Zhou J, Ding S, Du D, Wang J, Liu Y, Lin Y. Recent advances in single-atom nanozymes for colorimetric biosensing. TrAC Trends Anal Chem. 2023;168(2): Article 117280.

[191] Pratsinis A, Kelesidis GA, Zuercher S, Krumeich F, Bolisetty S, Mezzenga R, Leroux J-C, Sotiriou GA. Enzyme-mimetic antioxidant luminescent nanoparticles for highly sensitive hydrogen peroxide biosensing. ACS Nano. 2017;11(12):12210–12218. 29182310 10.1021/acsnano.7b05518

[192] Feng J, Li N, Du Y, Ren X, Wang X, Liu X, Ma H, Wei Q. Ultrasensitive double-channel microfluidic biosensor-based cathodic photo-electrochemical analysis via signal amplification of SOD-Au@PANI for cardiac troponin I detection. Anal Chem. 2021;93(42):14196–14203. 34636556 10.1021/acs.analchem.1c02922

[193] Gao X, Wei H, Ma W, Wu W, Ji W, Mao J, Yu P, Mao L. Inflammation-free electrochemical in vivo sensing of dopamine with atomic-level engineered antioxidative single-atom catalyst. Nat Commun. 2024;15(1):7915. 39256377 10.1038/s41467-024-52279-5 PMC11387648

[194] Bi J, Mo C, Li S, Huang M, Lin Y, Yuan P, Liu Z, Jia B, Xu S. Immunotoxicity of metal and metal oxide nanoparticles: From toxic mechanisms to metabolism and outcomes. Biomater Sci. 2023;11(12):4151–4183. 37161951 10.1039/d3bm00271c

[195] Wang X, Li Q-Z, Zhao Y, Gao X. Recent advances in oxidase-like nanozymes: Mechanisms, prediction models, and applications. ACS Appl Mater Interfaces. 2025;17(49):66110–66150. 41273330 10.1021/acsami.5c15196

[196] Liu W, Zhao N, Yin Q, Zhao X, Guo K, Xian Y, Li S, Wang C, Zhu M, Du Y, et al. Injectable hydrogels encapsulating dual-functional Au@Pt core–shell nanoparticles regulate infarcted microenvironments and enhance the therapeutic efficacy of stem cells through antioxidant and electrical integration. ACS Nano. 2023;17(3):2053–2066. 36695873 10.1021/acsnano.2c07436 PMC9933615

[197] Deng Z, Ma W, Ding C, Wei C, Gao B, Zhu Y, Zhang Y, Wu F, Zhang M, Li R, et al. Metal polyphenol network/cerium oxide artificial enzymes therapeutic nanoplatform for MRI/CT-aided intestinal inflammation management. Nano Today. 2023;53: Article 102044.

[198] He H, Xiong J, Pei L, Miao L, Song Y, Liu L, Wang L. An electrospun nanofibrous membrane dressing composed of gallium-curcumin nanoparticles encapsulated by covalent organic framework with antibacterial and antioxidant abilities for enhanced wound healing and recovery. Chem Eng J. 2025;525: Article 170467.

[199] Wu J, Yu Y, Cheng Y, Cheng C, Zhang Y, Jiang B, Zhao X, Miao L, Wei H. Ligand-dependent activity engineering of glutathione peroxidase-mimicking MIL-47(V) metal–organic framework nanozyme for therapy. Angew Chem Int Ed Engl. 2021;60(3):1227–1234. 33022864 10.1002/anie.202010714

